# HOPS-dependent vesicle tethering lock inhibits endolysosomal fusions and autophagosome secretion upon the loss of Syntaxin17

**DOI:** 10.1126/sciadv.adu9605

**Published:** 2025-06-06

**Authors:** Dávid Hargitai, Anikó Nagy, Iván Bodor, Győző Szenci, Hajnalka Laczkó-Dobos, Arindam Bhattacharjee, Natali Neuhauser, Szabolcs Takáts, Gábor Juhász, Péter Lőrincz

**Affiliations:** ^1^ Department of Anatomy, Cell and Developmental Biology, Eötvös Loránd University, Budapest, Hungary.; ^2^ HAS-ELTE Momentum Vesicle Trafficking Research Group, Eötvös Loránd University, Budapest, Hungary.; ^3^ Institute of Genetics, HUN-REN Biological Research Centre Szeged, Szeged, Hungary.; ^4^ Developmental Biology Group, Agharkar Research Institute, Pune, India.

## Abstract

The autophagosomal SNARE (soluble *N*-ethylmaleimide–sensitive factor attachment protein) Syntaxin17 (Syx17) plays a pivotal role in autophagosome-lysosome fusion, yet the broader impact of its loss remains elusive. Our investigation of Syx17 function in *Drosophila* nephrocytes and salivary gland cells revealed unexpected effects. We find that Syx17 loss induces the formation of autophagosome-lysosome clusters in a HOPS (homotypic fusion and vacuole protein sorting)–dependent manner, entrapping this tether, autophagosomes, and lysosomes. While locked in clusters, these organelles cannot participate in other vesicle fusions, impeding endosomal progression and autophagosome secretion. Therefore, the absence of Syx17 not only inhibits autophagosome-lysosome fusion but also prevents HOPS release from autophagosome-lysosome tethering sites causing a “tethering lock.” Preventing autophagosome formation or removing the HOPS adaptor Plekhm1 (pleckstrin homology domain–containing family M member 1) leads to release of HOPS and lysosomes from these clusters, thus rescuing secondary effects of Syx17 loss. Our findings show that a tethering lock can disrupt multiple vesicle trafficking routes.

## Teaser

Syntaxin17 loss causes HOPS-dependent “tethering lock,” disrupting multiple vesicle trafficking events.

## INTRODUCTION

The compartmentalization of eukaryotic cells enables precise control of separate functions and transport routes between organelles, which is primarily achieved through membrane fusion. Most vesicle fusions are SNARE (soluble *N*-ethylmaleimide–sensitive factor attachment protein)–dependent processes, during which specific membrane lipids and membrane-associated small guanosine triphosphatases (GTPases) recruit tethering factors that anchor the interacting membranes together. Subsequently, tether-integral or separate SM (Sec1/Munc-18) family proteins facilitate the assembly and zippering of SNARE complexes, ultimately leading to membrane fusion (*1*). SNARE complexes are typically formed by three or four SNARE proteins, each containing one or two of the eponymous motifs. Four classes of SNARE motifs are distinguished (Qa, Qb, Qc, and R) within the four-helix bundle (*2*). According to the 3Q:1R rule, all functional SNARE complexes contain one member of each class; thus, most SNARE complexes are assembled from four proteins (*3*, *4*). However, in the case of Qbc SNAREs that have two SNARE motifs, an additional Qa- and an R-SNARE are enough to form a functional complex (*4*).

The two primary pathways of lysosomal degradation are endocytosis and macroautophagy (hereafter called autophagy). Both endosomes and autophagosomes mature into degradative endo- and autolysosomes by fusing with lysosomes (*5*). Converging in the same organelle, these pathways share several molecular components. In animal cells, Rab7 and Rab2 (Ras-related in brain GTPases 7 and 2) on late endosomes and autophagosomes recruit the multisubunit HOPS (homotypic fusion and vacuole protein sorting) tethering complex via adaptor proteins such as RILP (Rab-interacting lysosomal protein), ORPL1 (oxysterol-binding protein-related protein 1L), FYCO1 (FYVE and coiled-coil domain autophagy adaptor 1), and PLEKHM1 (pleckstrin homology domain–containing family M member 1) to ensure their fusion with Rab2, Rab7, or Arl8 (adenosine diphosphate ribosylation factor–like GTPase 8)–positive lysosomes (*6*–*11*). This enables HOPS to anchor autophagosomes and endosomes either together or to lysosomes, promoting their fusion (*5*, *12*, *13*).

Syntaxin17 (Syx17 in flies, STX17 in mammals) functions as the autophagosomal Qa-SNARE essential for autophagosome-lysosome fusion in animal cells (*14*, *15*). STX17 is recruited to the autophagosomal membrane in a PI4P (phosphatidylinositol 4-phosphate)–dependent manner (*16*, *17*), where it forms a complex with the Qbc-SNARE SNAP29 (synaptosomal-associated protein 29 kDa) and one of the lysosomal R-SNARE proteins VAMP7 or VAMP8 (vesicle-associated membrane protein 7 or 8) to execute the fusion (*14*, *15*, *18*). In addition, in mammalian cells, other SNARE proteins are also involved in autophagosome-lysosome fusion, such as the Qa-SNARE STX7, Qbc-SNARE SNAP47 (synaptosomal-associated protein 47 kDa), and the R-SNARE YKT6 (*19*, *20*). However, in *Drosophila* and in some mammalian cell types, Ykt6 appears to function as a regulatory SNARE, facilitating the formation of the fusion-competent Syx17-containing complex (*21*, *22*). Since VAMP8 and SNAP47 are absent in flies, the Syx17-Snap29-Vamp7 complex appears to be the sole functional SNARE complex responsible for executing autophagosome-lysosome fusion in *Drosophila*. Besides its role in autophagosome-lysosome fusion, STX17 seems to be also involved in mitochondrial fusions and selective mitophagy (*23*–*25*).

Except for Syntaxin17, the evolutionary conserved players in autophagosome-lysosome fusion (Rab2, Rab7, Arl8, HOPS, Snap29, Vamp7, and Ykt6) are also involved in other lysosome trafficking processes, such as in endosomal fusions or biosynthetic transport to lysosomes (*9*, *10*, *12*, *26*–*39*). This makes Syntaxin17 unique as the loss of this SNARE did not cause a defect in endosomal trafficking in either fly fat tissue, developing eye, or mammalian cells (*26*, *40*), This observation suggests that Syntaxin17 is specific to autophagy within the lysosomal system, despite phylogenetic analysis placing it within the endosomal Qa-SNAREs group (*41*).

*Drosophila* larval garland nephrocytes serve as excellent model cells for investigating endosomal traffic due to their continuous high endocytic activity (*9*, *28*, *29*). We used these cells to characterize the Vps8 (vacuolar protein sorting 8)–containing miniCORVET (mini class C core vacuole/endosome tethering complex) complex that is essential for endosomal degradation only under conditions of high endocytic activity, a discovery that could have otherwise remained hidden (*29*). Since previous investigations focused on the roles of Syx17/STX17 in cells with relatively low endocytic activity (*26*, *42*), we sought to examine the involvement of Syx17 in endocytosis using fruit fly nephrocytes.

We observed an interesting phenotype: a defect in late endosome maturation. Further investigations revealed unexpected results: In Syx17-depleted cells, autophagosome-lysosome clusters appeared, and the autophagosomes failed to be either degraded or secreted. This phenotype was unique: Similar clusters were absent in other autophagosome-lysosome fusion–impaired cells, such as those lacking HOPS, Rab7, Snap29, or Vamp7, and these cells could still secrete undegraded autophagosomes. We found that the autophagosome-lysosome clusters in Syx17 loss-of-function cells accumulated HOPS, whereas mature endosomes displayed a loss of this tether from their membranes. Notably, upon removal of HOPS or its adaptor Plekhm1 from Syx17 knockdown (KD) cells, these clusters disappeared, and the number of autophagosomes decreased. In addition, in Plekhm1 and Syx17 double RNA interference (RNAi) cells, HOPS localization on endosomes was restored, and the lysosomes were not trapped into clusters, leading to complete rescue of the endosomal defect. This finding shows that the clusters entrapping HOPS and lysosomes are responsible for the endosomal defect in Syx17-deficient cells. Moreover, the impairment of autophagosome secretion can also be attributed to the inability of autophagosomes to escape from these clusters.

Our findings reveal a previously unrecognized phenomenon in which the absence of a single, specific component of a SNARE complex leads to persistent tethering of organelles, indirectly affecting cellular processes beyond its primary role. We propose to call this effect “tethering lock.”

## RESULTS

### Loss of Syx17 causes endosome maturation defect in an autophagosome-dependent manner

**Fig. 1.**
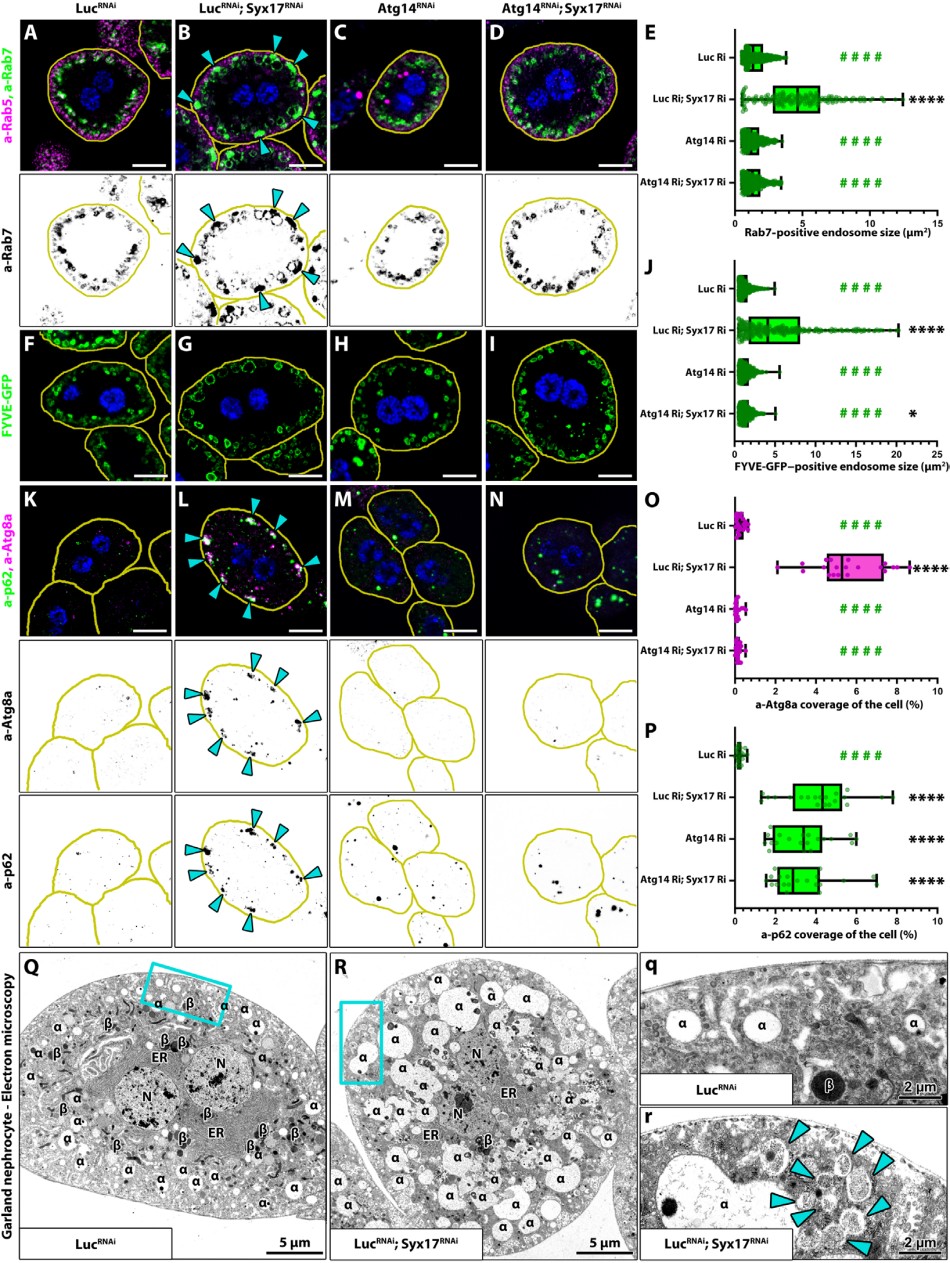
Syx17 RNAi induces endosome enlargement and the appearance of autophagosome containing vesicle clusters. Blue channel shows the nuclei of the cells in fluorescent images, and the outline of the cells is indicated by yellow lines. (**A** to **D**) Rab7-positive late endosomes are enlarged in Syx17 RNAi nephrocytes (B) compared to control (A) and Atg14 RNAi (C) cells. Coexpressing Atg14 RNAi with Syx17 RNAi restores Rab7-positive endosome size (D). Cyan arrows in (B) point to Rab7-positive aggregates. Scale bars, 10 μm. (**F** to **I**) GFP-2xFYVE–positive endosomes are enlarged in Syx17 RNAi nephrocytes (G) compared to control (F) and Atg14 RNAi (H) cells. Coexpressing Atg14 RNAi with Syx17 RNAi restores GFP-2xFYVE–positive endosome size (I). Scale bars, 10 μm. (**E** and **J**) Quantifications of Rab7- or FYVE-GFP–positive endosome sizes shown in (A) to (D) and (F) to (I), respectively. *n* = 174 to 476 endosomes from 10-10 cells (Rab7), *n* = 252 to 481 endosomes from 10-10 cells (FYVE-GFP). (**K** to **N**) Atg8a/p62-positive aggregates can be detected at the cell periphery of Syx17 RNAi nephrocytes [(L), cyan arrows] compared to control (K), Atg14 RNAi (M), or Atg14-Syx17 double RNAi (N) cells. Of note, compared to controls, more p62-positive puncta can be observed in Atg14 or Atg14-Syx17 double RNAi cells, indicating effective autophagosome formation inhibition. Scale bars, 10 μm. (**O** and **P**) Quantifications of Atg8a-positive (O) or p62-positive (P) areas shown in shown in (K) to (N). *n* = 20 cells. [(E), (J), (O), and (P)] Asterisks (*) indicate comparisons to the control, while green hashes (#) indicate comparisons to to Syx17-Luciferase double knockdown. ****P < 0.0001 and ####*P* < 0.0001 (**Q** and **R**) Ultrastructural analysis of nephrocytes revealed enlarged late endosomes (marked by α) and autophagosome [blue arrowheads in (r)] containing clusters in Syx17-depleted cells [(R) and (r)] compared to controls. β marks lysosomes.

Garland and pericardial nephrocytes, components of the insect renal system, exhibit traits similar to vertebrate renal glomerular podocytes and proximal tubule cells. These cells use a vertebrate-like slit diaphragm and endocytic scavenger receptors to filter hemolymph, storing or degrading the material they endocytose (*43*–*45*). Because of their persistent high endocytic activity, the endolysosomal compartment occupies a substantial portion of the cytoplasm: These vesicles form discernible layers within garland nephrocytes, making them ideal models to study endocytosis-related pathways (*9*, *28*, *29*). To analyze the potential involvement of *Drosophila* Syx17 in endosomal degradation, we first stained dissected nephrocytes with antibodies specific to early endosomal Rab5 and late endosomal Rab7 or examined 2xFYVE-GFP [2× Fab1, YOTB, Vac1, and EEA1 domain fused to green fluorescent protein: a PI3P (phosphatidylinositol 3-phosphate) reporter marking early autophagic membranes and endosomes] expressing cells. We detected enlarged late endosomes in both independent Syx17 RNAi (Fig. 1, A, B, E, F, G, and J, and fig. S1, A and G) and in *Syx17^LL^* mutant nephrocytes (fig. S1, D, E, and H). Notably, electron microscopy revealed that in Syx17 RNAi cells, the cytoplasm is filled with enlarged α vacuoles (late endosomes; Fig. 1, Q and R), while Rab7 fluorescence is primarily detected on their outermost layer (Fig. 1, A and B). This likely reflects a failure of endosomes to mature into endolysosomes, eventually leading to Rab7 dissociation and an arrested maturation state, closely resembling the late endosome maturation defects observed in cells depleted of HOPS, Rab2, or Arl8 (*9*, *10*, *28*, *29*). In addition, the accumulation of defective endosomes appears to reduce the space occupied by the endoplasmic reticulum (ER) (Fig. 1, Q and R), a phenomenon we confirmed by examining the pattern of the KDEL-RFP (lysine–aspartic acid–glutamic acid–leucine ER retention signal containing red fluorescent protein) ER reporter (fig. S1, I and J). The endosome maturation defect observed in *Syx17^LL^* mutant nephrocytes was rescued by expressing a Syx17-GFP transgene, confirming that the phenotype is specifically caused by the loss of *Syx17* (fig. S1, F and H).

Since defects in endosomal recycling may increase endosome size and alter nephrocyte lacunar channel depth or slit diaphragm morphology (*46*), we examined these structures by immunostainings, channel diffusion assays, and electron microscopy (fig. S1, L to U). However, we found no discernible difference between control and Syx17 RNAi cells, indicating that endosomal recycling proceeds normally (fig. S1, L to U). Consistently, silver nitrate (AgNO_3_) uptake—a commonly used tracer to assess filtering diaphragm function and detoxification capacity in fly nephrocytes (*43*, *44*, *47*)—proceeds normally in Syx17 RNAi nephrocytes (fig. S1, V and W).

We also investigated the localization of Syx17-GFP in nephrocytes. This reporter did not label endosomes (fig. S2A), and it was primarily observed on autophagosomes (fig. S2B), suggesting that its role in endolysosome function is indirect.

Furthermore, we observed an intriguing phenomenon: Syx17-depleted or mutant cells accumulated structures beneath their plasma membrane, showing a more intense Rab7 signal than late endosomes underneath [indicated with cyan arrows in Fig. 1B and fig. S1 (A and E)]. Similar structures were not observed in 2xFYVE-GFP–expressing Syx17-depleted cells (Fig. 1G), suggesting a non-endosomal origin. Given that myotubularins remove PI3P from maturing autophagosomes and considering that mutant *Drosophila* fat cells lacking Syx17 accumulate autophagosomes (*15*, *48*–*50*), we hypothesized that these structures represent autophagosomes. This was further supported by the detection of similar compact structures in Atg8a (autophagy-related 8a) and p62/ref2P (refractory to SigmaP) immunostained RNAi or mutant samples (Fig. 1, K, L, O, and P, and fig. S2, C, F, G, and I to L), and electron microscopy revealed the presence of autophagosome clusters beneath the plasma membrane of Syx17 RNAi cells, which was not seen in controls (Fig. 1, Q, q, R, and r). Notably, the Atg8a- and P62/ref2P-positive aggregates were completely absent in rescued mutant cells (fig. S2, F to H, K, and L).

The autophagic origin of the peripheral clusters was further confirmed by the fact that Atg14 or Atg1 RNAis (which had no obvious effect on endosomes) effectively eliminated them from Syx17 RNAi cells (Fig. 1, M to P, and fig. S2, D, E, I, and J). The expression of both Atg RNAis had an intriguing secondary effect: They were also able to rescue the late endosomal phenotype induced by Syx17 RNAi (Fig. 1, C to E and H to J, and fig. S1, B, C, and G), suggesting a connection between the late endosome maturation defect and the accumulation of autophagosomes.

We have previously investigated nephrocytes in which known factors involved in autophagosome-lysosome fusion were mutated or depleted, but we have never observed autophagosome clusters in them (*9*, *10*, *28*, *29*). However, we have never compared their phenotypes to each other. Thus, we depleted Snap29 and Vamp7 SNARE proteins, HOPS (Vps41 or Vps11) subunits, and small GTPases (Rab2, Rab7, and Arl8) in nephrocytes using RNAi and stained the samples with anti-Atg8a and anti-Ref2p/p62 (fig. S3). As expected, given their involvement in autophagosome-lysosome fusion, in most cases, we detected an elevation of Atg8a and p62-positive puncta. However, in HOPS, small GTPase, or Vamp7-depleted cells, the autophagosome or p62 accumulation was considerably less pronounced than in Syx17 RNAi cells, and autophagosome clusters were not present within them. Of note, by using one Snap29 RNAi line, we sometimes detected autophagosome clusters (fig. S3C), but garland nephrocytes were completely devoid of such structures in another two independent Snap29 RNAi lines (fig. S3, D and E). These results suggest that the formation of autophagosome clusters is specific for Syx17 loss; therefore, we next examined these structures in detail.

### HOPS-tethered autophagosome-lysosome clusters form in Syntaxin17-depleted *Drosophila* and human cells

**Fig. 2.**
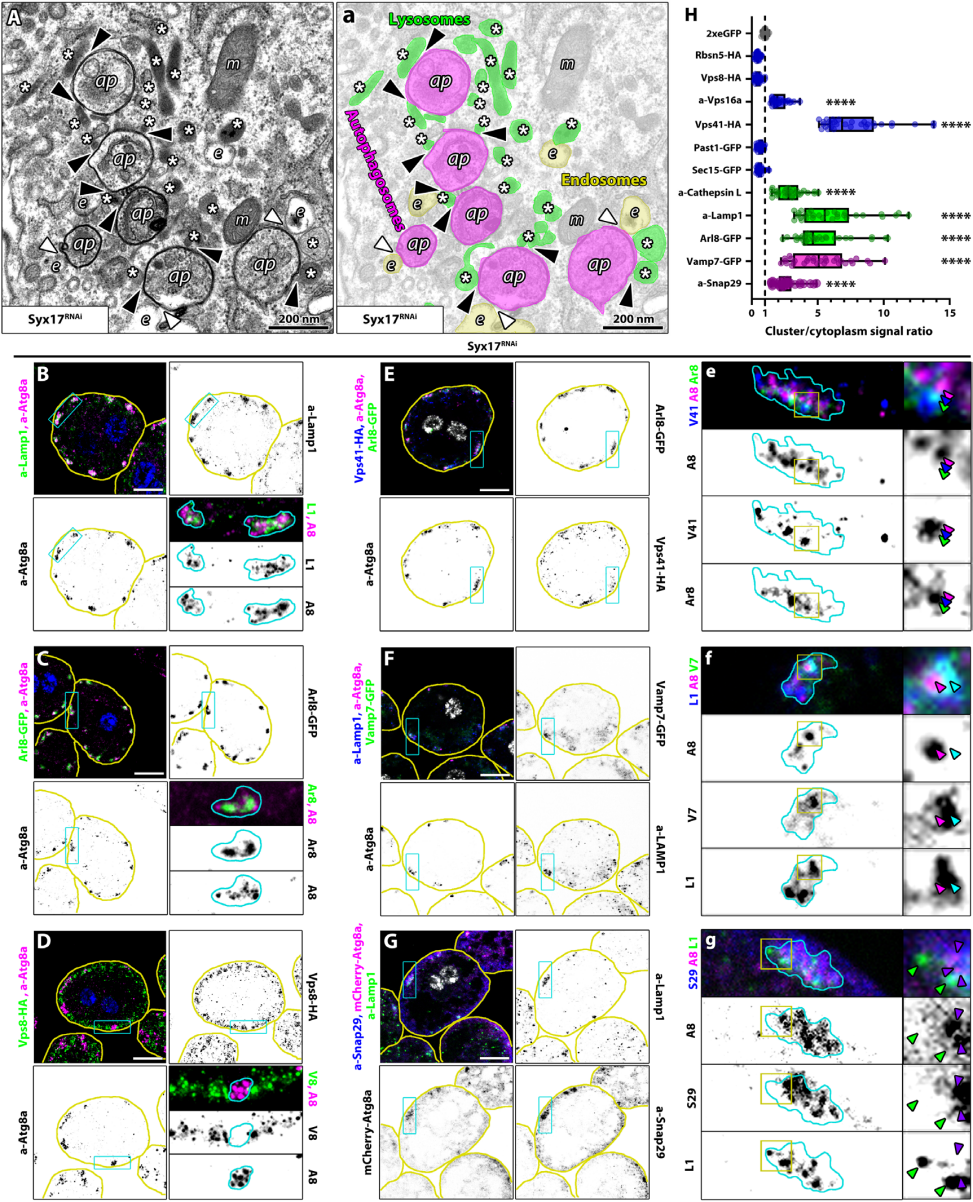
The autophagosome clusters in Syx17-depleted cells also contain lysosomes and are positive for Vamp7, Snap29, and the HOPS complex. (**A**, a) Ultrastructure of the clusters in Syx17 RNAi cells: Lysosomes (asterisks) and some late endosomes (e) were found in the close vicinity of autophagosomes (ap). White and black arrowheads point to endosome-autophagosome and lysosome-autophagosome contacts, respectively. m, mitochondria. (a) False-color overlay was added to (A) to help visualization of organelles and contacts. (**B** to **G**) The outlines of the cells and autophagosome clusters are indicated by yellow and cyan lines, respectively. Scale bars, 10 μm. [(B) and (C)] Fluorescent imaging revealed Lamp1 (B)– and Arl8-GFP (C)–positive structures, in the clusters of Syx17-depleted cells, showing minimal overlap with Atg8a^+^ autophagosomes. Blue channel shows the nuclei of the cells. [(D), (E), and (e)] The HOPS-specific reporter Vps41-HA [(E) and (e)] is detected in clusters on Arl8-GFP–positive structures (green arrow) or at the interface between Arl8-GFP and Atg8a-positive structures (blue arrow). Atg8a-positive structures (magenta arrow) are rarely positive for Vps41-HA. In contrast, the clusters are not positive for miniCORVET-specific Vps8-HA (D). [(F) and (f)] Vamp7-GFP is detected in the clusters, showing overlap with Lamp1 (cyan arrows) but not with Atg8a (magenta arrow). The white channel in the composite image represents the nuclei of the cells. [(G) and (g)] Endogenous Snap29 is detected in the clusters, showing overlap with Atg8a-mCherry (purple arrows) but not with Lamp1 (green arrow). The white channel in the composite image represents the nuclei of the cells. Since Snap29 is also reported to be required for endocytosis (*37*), it could also be detected within the layer of early endosomes [(E) and (e)]. (**H**) Quantification of cluster enrichment data, incorporating results from (B) to (G) and fig. S3 (B to G). Asterisks (*) indicate comparisons to the control. ****P < 0.0001. *n* = 30.

Ultrastructural analyses revealed that small electron-dense structures, presumably lysosomes, can be found in very close vicinity or directly next to the autophagosomes accumulating in Syx17 RNAi cells (Fig. 2, A and a, and fig. S4, A and a). Fluorescent microscopy confirmed that the Atg8a-positive clusters also contained structures positive for lysosomal markers Lamp1 (lysosomal-associated membrane protein 1), Arl8-GFP, and CathL (cathepsin L), with minimal or no overlap with Atg8a (Fig. 2, B, C, and H, and fig. S4, B and C). Given that the clusters identified through ultrastructural analysis contain autophagosomes, lysosomes, and occasionally endosomes in close proximity, we hypothesized that these organelles are tethered together. Supporting this, we detected the HOPS-specific Vps41–hemagglutinin (HA) reporter and the class C core protein Vps16A within these structures (Fig. 2, E and H, and fig. S4D). Notably, Vps41-HA was predominantly observed on lysosomes or in the space between lysosomes and autophagosomes (Fig. 2e). In contrast, when reporters specific to other tethering factors of the endosomal system were expressed [such as Rabenosyn-5-HA/Rbsn5-HA, miniCORVET-specific Vps8-HA, exocyst-subunit secretory (Sec) 15-GFP, or the supposedly FERARI (factors for endosome recycling and Rab interactions)–subunit Past1-GFP (protein associated with star-like trichome 1), the fly ortholog of human EHD1 (EH domain–containing protein 1)] (*51*), their signals were not enriched within the clusters (Fig. 2, D and H, and fig. S4, E to G). Since Syx17 forms a complex with Snap29 and Vamp7 during autophagosome-lysosome fusion, we also examined the localization of Vamp7-GFP and endogenous Snap29 in Syx17 KD cells. Both were detected in the clusters (Fig. 2, F to H). Notably, Vamp7-GFP signal primarily originated from lysosomes, while Snap29 was observed on autophagosomes (Fig. 2, f and g). As Q-SNAREs typically localize to one of the interacting membranes and R-SNAREs to the other (*4*, *52*), our findings align with the known roles of these proteins. Vamp7, a well-characterized late endosomal/lysosomal R-SNARE, localizes to lysosomes in the clusters. As Syx17, a Qa-SNARE, resides on autophagosomes (*14*, *15*, *36*), it follows that Snap29, a Qbc-SNARE, should also localize to autophagosomes, which is precisely what we observed.

**Fig. 3.**
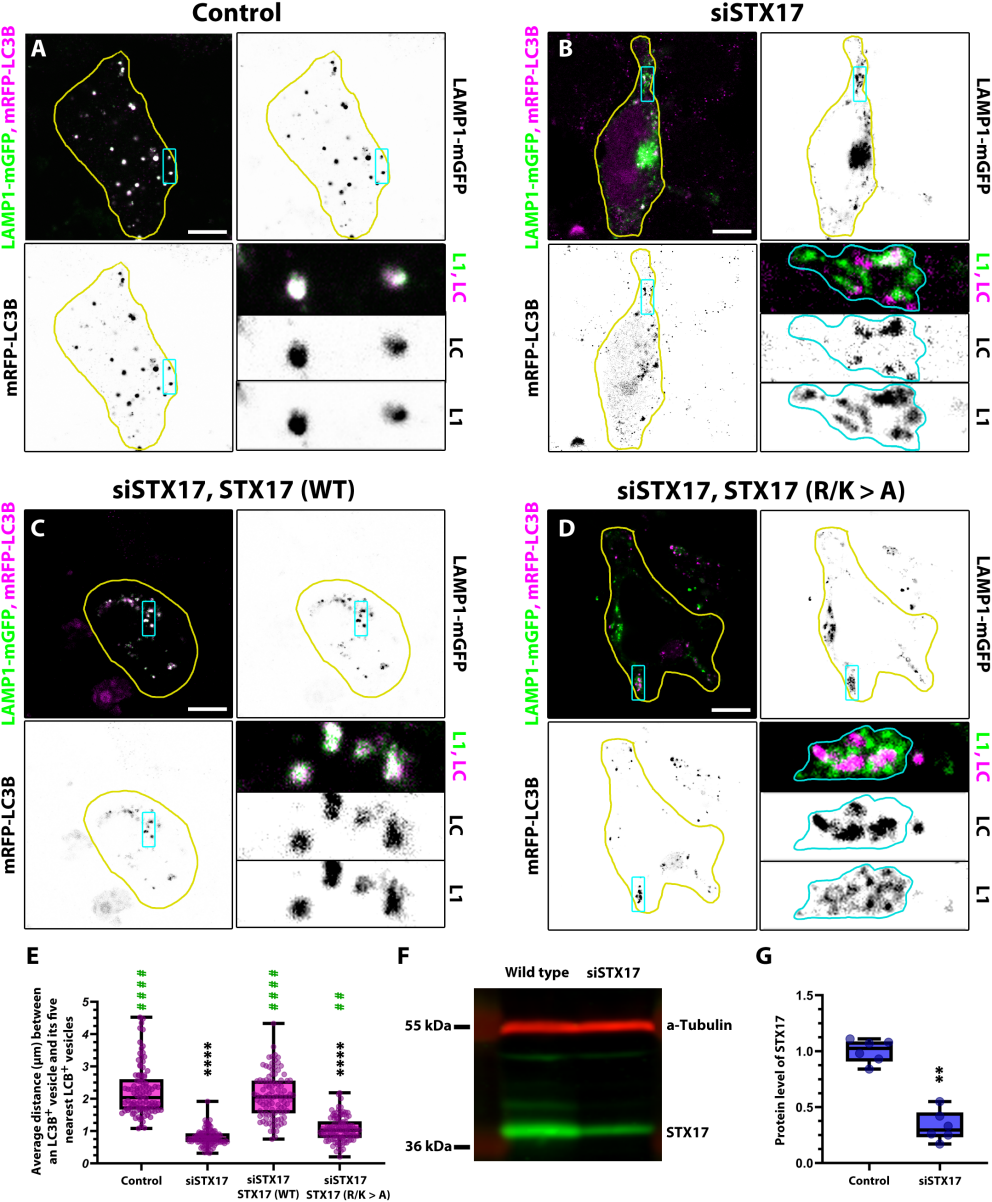
Autophagosome-lysosome clusters in cultured and starved mammalian STX17 KD cells. STX17 was knocked down in HEK-293 cells, which were then transfected with siRNA-resistant forms of wild-type (WT) or R/K > A mutant STX17, mRFP-LC3B, and LAMP1-mGFP. (**A** and **C**) In control and rescued cells, the mRFP-LC3B and LAMP1-GFP signals overlap, indicating that autophagosome-lysosome fusion and autolysosome formation proceed normally. (**B** and **D**) In contrast, in STX17 KD cells and STX17 KD cells expressing mutant STX17, the signals do not overlap, indicating inhibited autophagosome-lysosome fusion. The reporter signals are often confined to clusters, indicating that autophagosomes and lysosomes are tethered together. The outlines of the cells and clusters are indicated by yellow and cyan lines, respectively. Scale bars, 10 μm. (**E**) Quantification of autophagosome-autophagosome (mRFP-LC3B^+^ Lamp1-mGFP^−^) distances in (A) to (D). *n* = 100. Green asterisks (*) indicate comparisons to siSTX17 RNAi, and black asterisks (*) indicate comparisons to control ****P < 0.0001 and **P < 0.01. (**F**) Western blot analysis confirming efficient STX17 knockdown. (**G**) Quantification of the Western blot data shown in (F). *n* = 6, **P < 0.01.

The role of Syx17 in autophagosome-lysosome fusion is conserved across animals, leading us to investigate whether similar structures form in STX17 KD human embryonic kidney (HEK) 293 cells. Clustered vesicles were observed in siSTX17 cells (Fig. 3, A, B, and E to G). Notably, this clustering effect was rescued when cells were transfected with small interfering RNA (siRNA)–resistant wild-type STX17, as Lamp1/LC3B (microtubule-associated protein 1A/1B-light chain 3B) double-positive autolysosomes appeared, resembling the control phenotype (Fig. 3, C and E). However, transfection with an siRNA-resistant STX17 containing Ala substitutions of Lys and Arg amino acid residues near its transmembrane (TM) domains failed to rescue the effect, and the clusters persisted (Fig. 3, D and E). This mutation leaves the N-terminal Habc and SNARE domains intact but disrupts the autophagosomal recruitment of STX17 (*16*), indicating that the efficient rescue of the fusion defect and the clustering effect both requires STX17 localization to the autophagosomal membrane.

### Autophagosomes fail to be secreted in the absence of Syx17

**Fig. 4.**
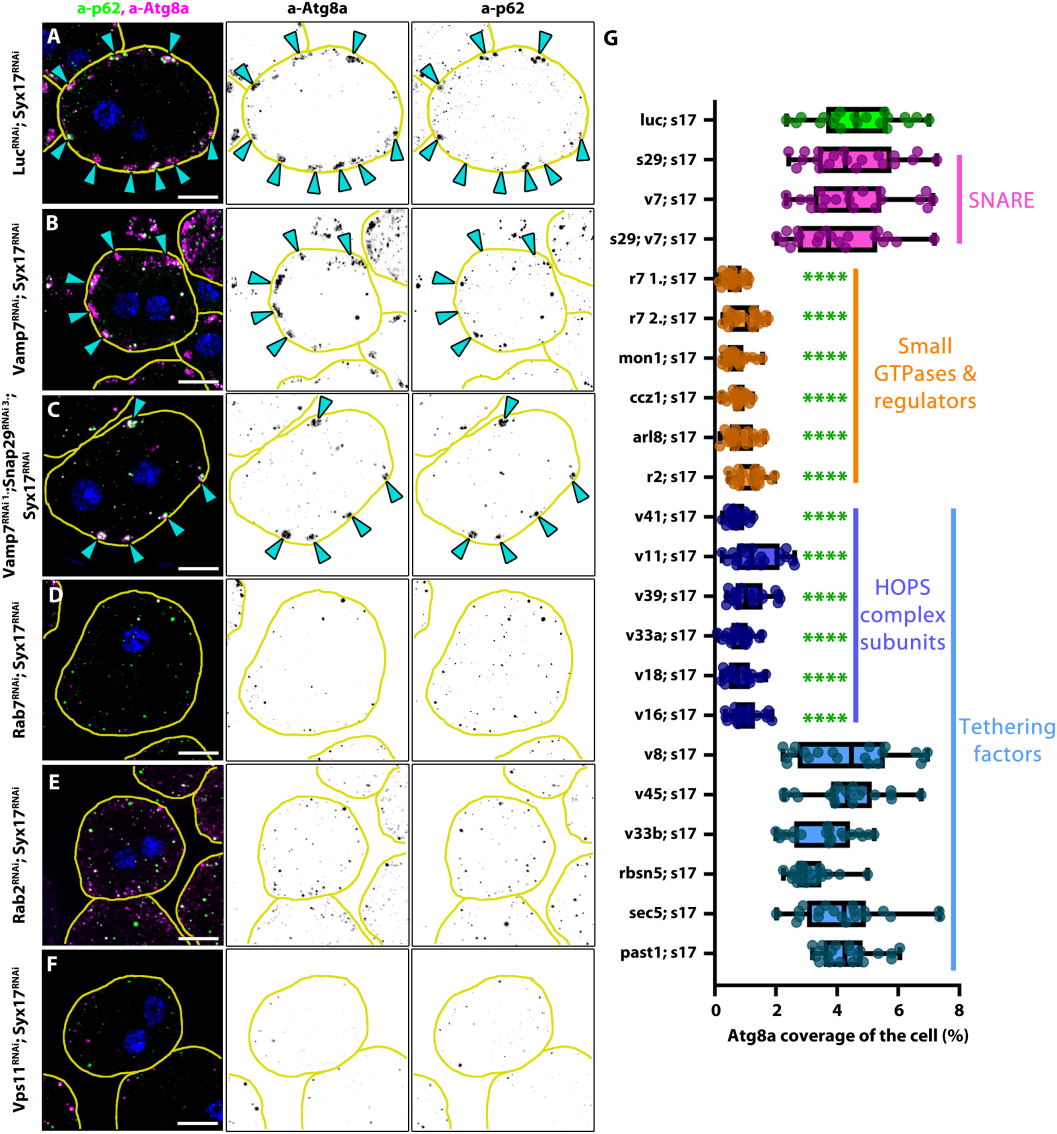
Autophagosome cluster formation in Syx17 RNAi cells depends on HOPS, Rab7, and Rab2. (**A** to **F**) Unlike Vamp7 RNAi (B) or Vamp7 with Snap29 RNAi (C), depletion of Rab7 (D), Rab2 (E), or the HOPS-specific subunit Vps11 (F) prevents autophagosome cluster formation in Syx17 RNAi cells. Cyan arrowheads point to autophagosome clusters in (A) to (C). Blue channel shows the nuclei of the cells [stained by 4′,6-diamidino-2-phenylindole (DAPI)]. The outline of the cells is indicated by yellow lines. Scale bars, 10 μm. (**G**) Quantification of Atg8a coverage data in (A) to (F) and in fig. S5 (A to P), *n* = 20 cells. Green asterisks (*) indicate comparisons to Syx17 RNAi. ****P < 0.0001.

Since autophagosomes appear to be tethered to lysosomes and endosomes in the clusters, we aimed to identify the factors involved in cluster formation by testing the epistatic relationship between Syx17 and other autophagosome-lysosome fusion components in fly nephrocytes. We found that clusters could appear in double (Syx17-Snap29 or Syx17-Vamp7) or triple (Syx17-Snap29-Vamp7) SNARE RNAi nephrocytes (Fig. 4, A to C, and fig. S5A), with the number of autophagosomes similar to that observed in Syx17 single RNAi cells (Fig. 4G). Conversely, Syx17-HOPS or Syx17–small GTPase (Rab7, Rab2, and Arl8) or Rab7 GEF (guanine nucleotide exchange factor) double RNAi cells showed no signs of such structures (Fig. 4, D to F, and fig. S5, B to J). Furthermore, we also observed an unexpected phenomenon: The number of autophagosomes reduced in these cases (Fig. 4G). In addition, autophagosome clusters remained present in Syx17 RNAi cells in which tethering factors unrelated to HOPS (such as Rbsn-5, Vps45, Vps8/miniCORVET, Sec5/exocyst, Past1/FERARI, and Vps33B) were also depleted (Fig. 4G and fig. S5, K to P).

These results suggest that the formation of the clusters is HOPS and small GTPase dependent, but on the basis of the triple RNAi experiment (Fig. 4C), it is SNARE independent. However, the question of why the number of autophagosomes is elevated in Syx17-depleted cells compared to others was puzzling. The observation that the level of endogenous p62 was also the highest in Syx17 single RNAi cells compared to others (fig. S3N) suggests that apart from Syx17 KD, nephrocytes can still dispose of materials typically degraded by autophagy through alternative pathways when autophagosome-lysosome fusion is blocked.

**Fig. 5.**
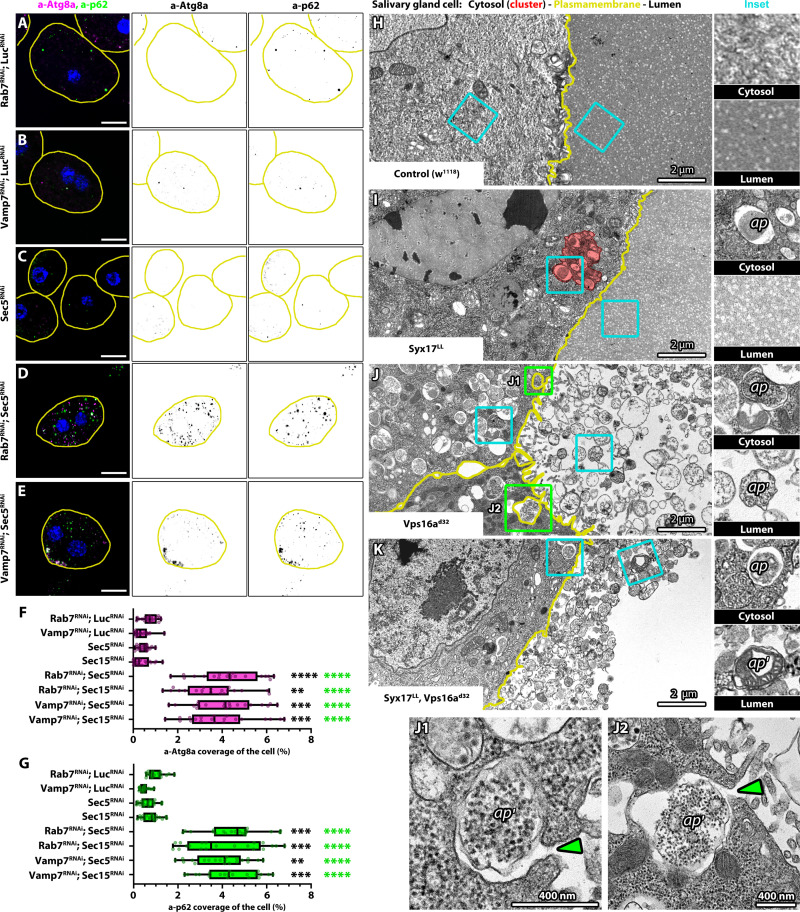
Undegraded autophagosome accumulates in exocyst and Vamp7 or Rab7 double RNAi cells, and the secretion failure in Syx17 RNAi cells depends on HOPS. (**A** to **E**) More Atg8a- and p62-positive puncta can be observed in Rab7-Sec5 and Vamp7-Sec5 double RNAi nephrocytes than in Rab7-Luciferase, Vamp7-Luciferase double, or Sec5 single RNAi controls. (**F** and **G**) Quantification of Atg8a and p62 coverage data in (A) to (E) and fig. S6 (A to C) (*n* = 20 cells). Green asterisks (*) indicate comparisons to Luc-Vamp7 double RNAi, while black asterisks (*) indicate comparisons to Lu-Rab7 double RNAi. (**H** and **I**) Ultrastructure of salivary glands: autophagosomes (a) are absent from the gland lumen and cytoplasm of gland cells of controls (H). Autophagosomes could be seen in the cytoplasm of gland cells of *Syx17^LL^* mutants, while no autophagic bodies (a′) were detected in the lumen (see insets on the right). The outline of an autophagosome cluster in (I) is indicated by a red line and a red overlay. This cluster is also shown at higher magnification in fig. S4Q. Secreted autophagic bodies could be detected in the salivary gland lumen of *Vps16^d32^* single (**J**) and *Syx17^LL^, Vps16a^d32^* double mutants (**K**) and the cytoplasm of mutant gland cells. Green insets (J1 and J2) show autophagosomes undergoing fusion with the plasma membrane, and green arrowheads point to the fusion pore.

Previous studies suggested that Snap29-depleted *Drosophila* imaginal discs or cultured mammalian cells impaired in autolysosome formation secrete undegraded autophagosomes (*37*, *53*–*55*). This led us to hypothesize that nephrocytes can secrete autophagosomes if autophagosome-lysosome fusion is blocked, except when Syx17 is depleted. Given that the exocyst complex is the main tether in secretory pathways and exocytosis (*56*), this suggests the possibility that nephrocytes secrete undegraded autophagosomes in an exocyst-dependent manner. Consistent with this, coexpressing exocyst subunit RNAi with Rab7 or Vamp7 RNAi resulted in a greater accumulation of autophagosomes in nephrocytes compared to Rab7 or Vamp7 single RNAi cells (Fig. 5, A to G, and fig. S6, A to C). Notably, since Rab7 is required for autophagosome transport (*57*), autophagosomes in Rab7-exocyst double RNAi cells remain dispersed. In contrast, in Vamp7-exocyst double RNAi cells, autophagosomes accumulate at the plasma membrane but fail to be secreted. Ultrastructural analysis further supports this, revealing autophagosomes near the plasma membrane in Vamp7-exocyst double RNAi cells. However, unlike in Syx17-depleted cells, these autophagosomes do not form clusters (fig. S6, D and d). In addition, lacunae were nearly absent in Vamp7-Sec5 RNAi nephrocytes, consistent with previous reports that exocyst silencing disrupts lacunar channel formation (*58*).

Nephrocytes float in the anterior body cavity of the larva and are surrounded by the blood, so these cells are not ideal for following secreted autophagosomes. We thus turned to salivary glands, which have a well-defined lumen. The salivary gland lumen of control and *Syx17*-null mutant animals is devoid of secreted autophagosomes, unlike that of *Vps16a^d32^* single or *Syx17^LL^*, *Vps16a^d32^* double-null mutants, which contain numerous autophagosomes (Fig. 5, H to K). Clusters of autophagosomes and lysosome-like vesicles were also detected in *Syx17^LL^* mutant salivary gland cells as in nephrocytes (Fig. 5I and fig. S6, E, e, F, and f). This indicates that Syx17-depleted cells are incapable of secreting autophagosomes, but this can be rescued by inhibition of autophagosome-lysosome tethering.

### Formation of autophagosome-lysosome clusters depends on the HOPS interactor Plekhm1 in Syx17-depleted cells

If autophagosomes are locked into clusters, then it is reasonable to conclude that, while tethered, they cannot escape and be secreted. This is why HOPS depletion rescues this effect, as, in this case, autophagosomes are not confined to clusters. Since the clusters did not appear upon inhibition of autophagosome formation and endosome size normalized (Fig. 1, A to E and K to P, and figs. S1, B, C, and G, and S2, D, E, I, and J), we hypothesized that the formation of these structures is related to the endosome maturation defect. As the autophagosome secretion defect was alleviated by releasing autophagosomes from the clusters through HOPS depletion (Fig. 5, H to K), it logically follows that releasing lysosomes and the HOPS tethering complex itself should restore the endosome maturation defect. However, HOPS, Rab2, Rab7, or Arl8 depletion all lead to an endosome maturation defect due to their involvement in endosome-lysosome fusion as well (*9*, *10*, *28*, *29*), so their loss of function is inadequate to test this hypothesis.

**Fig. 6.**
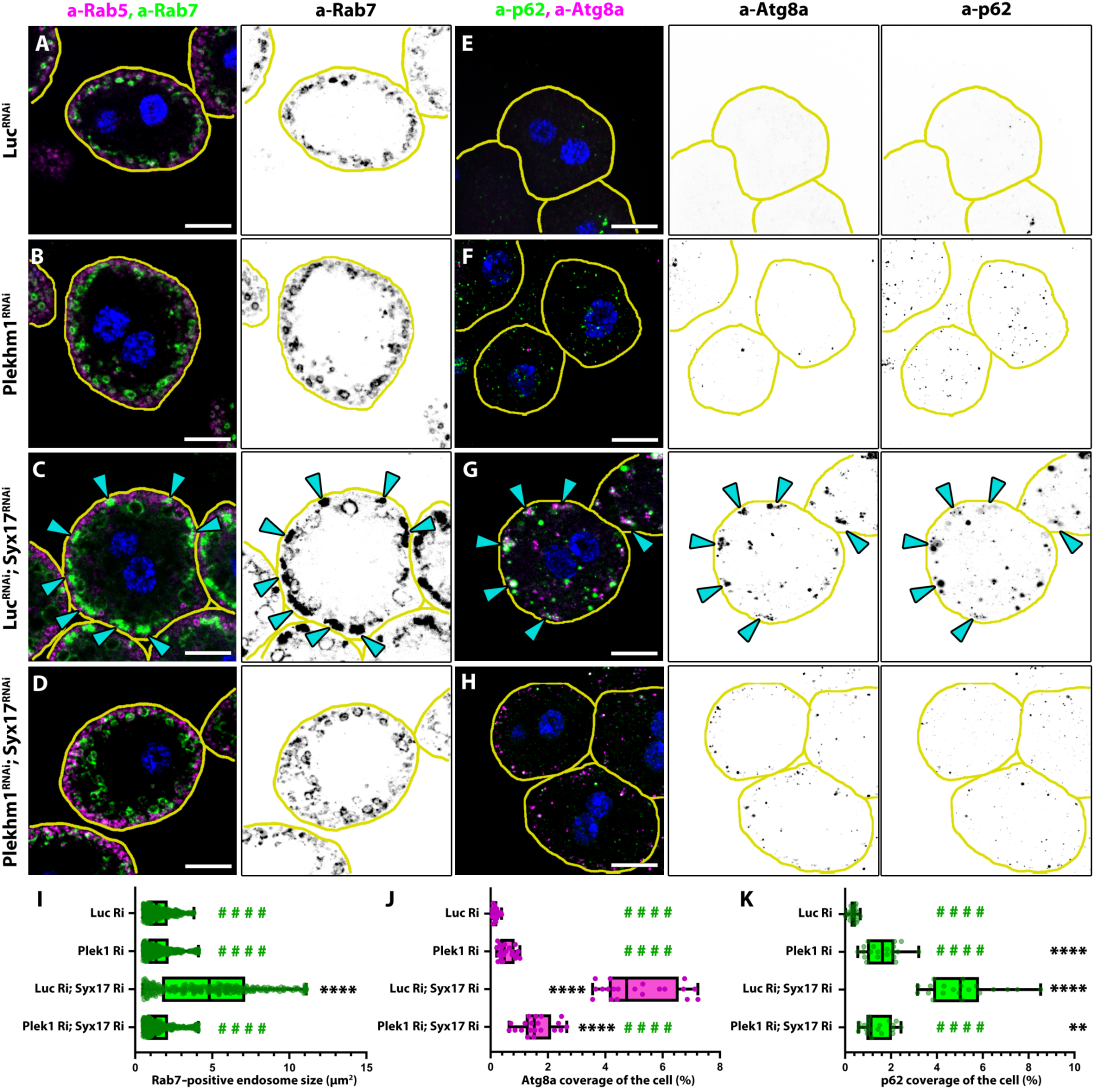
Depletion of Plekhm1 in Syx17 RNAi cells restores endosome size and eliminates autophagosome clusters. (**A** to **D**) Rab7-positive late endosomes are normal in Plekhm1 RNAi nephrocytes (B) compared to controls (A), and expression of Plekhm1 RNAis in Syx17 RNAi cells (D) restores endosome size compared to control (Luciferase-) and Syx17 RNAi coexpressing cells (C). Blue channel shows the nuclei of the cells (stained by DAPI). The outline of the cells is indicated by yellow lines. Cyan arrows in (C) point to Rab7-positive aggregates. Scale bars, 10 μm. (**E** to **H**) Atg8a/p62-positive clusters cannot be observed in Plekhm1 single RNAi or Plekhm1-Syx17 double RNAi cells [(F) and (G)] in contrast to control (Luciferase-) RNAi coexpressing Syx17 RNAi cells (H). Notably, an elevated number of Ref2P/p62 puncta can be observed in all RNAi cells (with the highest number coming from Luciferase-Syx17 double RNAi cells) compared to control RNAi expressing cells, indicating a failure in autophagic degradation. Blue channel shows the nuclei of the cells (stained by DAPI). The outline of the cells is indicated by yellow lines. Cyan arrows in (G) point to Atg8a-positive autophagosome clusters. Scale bars, 10 μm. (**I** to **K**) Quantifications of data shown in (A) to (H). Asterisks (*) indicate comparisons to the control (Luciferase single RNAi), while green hashes (#) indicate comparisons to Luciferase-Syx17 double knockdown. **P < 0.01, ****P < 0.0001, and ####*P* < 0.0001. (I) Quantification of Rab7-positive endosome size in (A) to (D), *n* = 278 to 474 endosomes from 10-10 cells. [(J) and (K)] Quantifications of Atg8a and p62 coverage in (E) to (H), respectively, *n* = 20 cells.

Therefore, we sought to identify a factor required for autophagosome-lysosome tethering without affecting endosome maturation. In animal cells, Rab7 indirectly binds to HOPS via several adaptor proteins (*6*–*11*). Among these, Plekhm1 and Def8 (defective in endocytic function 8) have been shown to interact with Rab7 in flies (*9*, *59*), but neither is essential in *Drosophila*, as their null mutants are viable (*60*). RNAi lines targeting Plekhm1 or Def8 have been shown to cause a mild autophagy defect, suggesting that these can promote HOPS-dependent autophagosome-lysosome fusion but are not essential for HOPS function (*60*). We thus examined Plekhm1 and Def8 loss of function in nephrocytes. Plekhm1 RNAi or its mutation had no discernible effect on endosome size or distribution, unlike Def8 RNAi, which resulted in the enlargement of late endosomes, indicating its involvement in endosome maturation (Fig. 6, A, B, and I, and fig. S7, A, B, E, G, M, and N). Furthermore, late endosomes were still able to acquire HOPS in Plekhm1 RNAi cells, suggesting that Plekhm1 is not required for HOPS recruitment onto endosomes (fig. S7, C and D), but as the number of p62 puncta increased, it seemed to be involved in autophagy (Fig. 6, E, F, and K). As suspected, in Plekhm1-Syx17 double RNAi and in *Plekhm1^d18^; Syx17^LL^* double mutant cells, no autophagosome clusters were detected (Fig. 6, E to H, J, and K, and fig. S7, I to L, O, and P), and late endosome size was restored to normal compared to single Syx17 RNAi or mutant nephrocytes (Fig. 6, C, D, and I, and fig. S7, E to H and N).

**Fig. 7.**
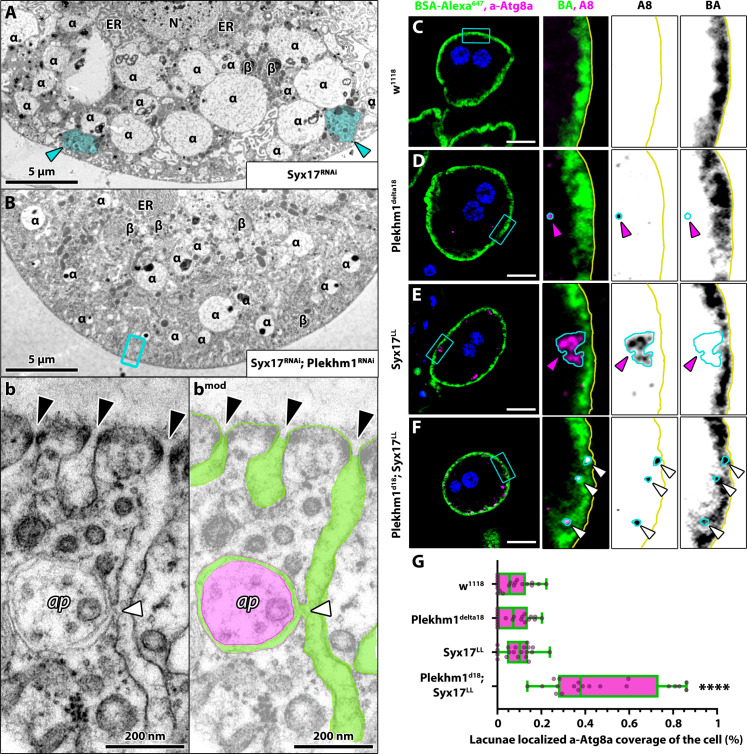
Autophagosomes are secreted in Plekhm1-Syx17 double KD nephrocytes. (**A** and **B**) Ultrastructural analysis of Syx17 RNAi (A) and Plekhm1-Syx17 double RNAi cells (B) revealed that the size of late endosomes (marked by α) is restored upon coexpression of Plekhm1 RNAi with Syx17 RNAi. Autophagosome-containing clusters are not present in double RNAi cells (B) compared to Syx17 single RNAi cells (A). Cyan arrowheads indicate autophagosome clusters, and a cyan overlay aids in identifying them in (A). (b) In double RNAi cells, we observed an autophagosome fusing with the lacunar plasma membrane, indicating that autophagosome secretion can occur in these cells. A green arrowhead points to the fusion pore. AP, autophagosome. A false-color overlay aids visualization of the fusion in (b^mod^). Green overlay shows the lumen of the lacunar channels, and magenta overlay shows the autophagic body of the secreted autophagosome. Black arrowheads point to lacuna openings sealed by slit diaphragms in (b) and (b^mod^). (**C** to **F**) Atg8a^+^ structures, presumably autophagosomes, are detected in the lacunae of *Plekhm1^d18^; Syx17^LL^* double-mutant nephrocytes (F) but not in wild-type controls (C) or *Plekhm1^d18^* and *Syx17^LL^* single mutants [(D) and (E), respectively], as shown by channel diffusion assays using BSA–Alexa Fluor 647 combined with Atg8a immunostaining. White arrowheads in (F) indicate Atg8a^+^ structures colocalizing with BSA–Alexa Fluor 647. Magenta arrowheads highlight an autophagosome beneath the plasma membrane in (D) and an autophagosome cluster under the plasma membrane in (E). (**G**) Quantification of data in (C) to (F), *n* = 20 cells.

These observations were further confirmed by ultrastructural analyses (Fig. 7, A and B), which revealed that the size of late endosomes is restored in Plekhm1-Syx17 double RNAi cells, and autophagosome-containing clusters are not present within them compared to Syx17 single RNAi cells. We also observed direct fusion between an autophagosome and the plasma membrane at the lacunar channel in double RNAi nephrocytes (Fig. 7, b and b^mod^), indicating that nephrocytes can remove autophagosomes from their cytosol through secretion when autophagosome-lysosome fusion is inhibited. This was further supported by channel diffusion assays complemented with Atg8a immunostainings. We observed Atg8a-positive puncta in the lacunar channels of *Plekhm1^d18^; Syx17^LL^* double mutant nephrocytes, which were rarely seen in controls (Fig. 7, C to G). Consistently, we observed individual autophagosomes in the salivary gland lumen of *Plekhm1^d18^; Syx17^LL^* double mutants (fig. S8, A and a), indicating that, similar to nephrocytes, double mutant salivary gland cells also secrete autophagosomes. Notably, autophagosomes appeared more abundant in the cytosol and gland lumen of *Vps16A^d32^* single or *Syx17^LL^, Vps16a^d32^* mutants (Fig. 5, J and K), consistent with the observation that *Vps16A^d32^* mutation enhances autophagosome formation by reducing Tor activity in flies (*42*).

Since the entrance of lacunar channels are sealed by slit diaphragms (*43*, *45*), we examined slit diaphragm and channel morphology of autophagosome secreting (*Plekhm1^d18^; Syx17^LL^* double mutant) nephrocytes. We found no discernible difference between the slit diaphragm and lacunar channel morphology of control and double mutant cells, indicating that secreted autophagosomes do not damage these structures (fig. S8, B to J).

### Releasing HOPS and lysosomes from the clusters rescues Syx17 KD induced endolysosomal defects

The increase of late endosome size suggests a late endosome maturation and lysosome fusion defect in Syx17-depleted cells; however, this phenotype alone cannot be used to determine whether endolysosomal degradation proceeds in these cells. The rapid endosomal traffic in nephrocytes transports endocytosed cargo to lysosomes roughly in 30 min, where it will be degraded (*61*). This enabled us to design a pulse-chase degradation assay to examine endolysosome function in nephrocytes (fig. S9A). We found that endolysosomes cannot degrade cargo properly in Syx17 RNAi cells, but this effect was rescued by inhibiting autophagosome formation or tethering by Atg14 or Plekhm1 depletion, respectively (fig. S9).

**Fig. 8.**
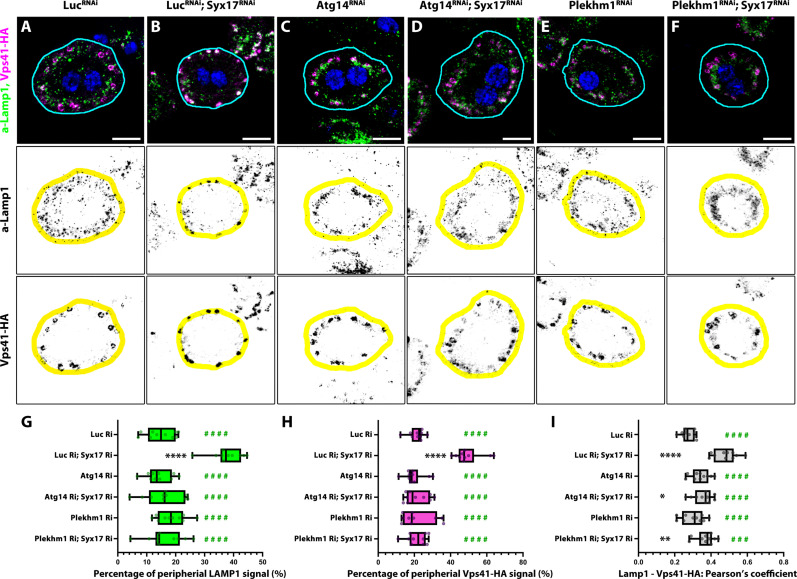
Atg14 or Plekhm1 depletion releases lysosomes and HOPS complex from the periphery of Syx17 RNAi cells. (**A** to **F**) Lysosomes and HOPS-positive vesicles in nephrocytes. In control nephrocytes (A), lysosomes appear as Lamp1-positive granules far from the periphery in a layer situated slightly below or overlapping with the late endosomal layer in which several Vps41-HA (HOPS)–positive endosomes can be detected. In Syx17 RNAi cells, most of the Lamp1 and Vps41-HA signals originate from the clusters found at the periphery of the cells, while almost no Vps41-HA–positive endosomes and only a few Lamp1 lysosomes can be detected in their respective layers. In contrast, the layout of Lamp1 and Vps41-HA seems control-like in Atg14 RNAi (C), Atg14-Syx17 double RNAi (D), Plekhm1 RNAi (E), and Plekhm1-Syx17 double RNAi (F) cells. Blue channel shows the nuclei of the cells (stained by DAPI). The outline of the cells is indicated by cyan lines. We defined a region as peripheral if it lies within the area shown by the yellow overlay (1.5-μm deep region). Scale bars, 10 μm. (**G** to **I**) Quantifications of data shown in (A) to (F). Asterisks (*) indicate comparisons to the control (Luc RNAi), while green hashes (#) indicate comparisons to Luc-Syx17 double RNAi. *P < 0.05, **P < 0.01, ****P < 0.0001, ###*P* < 0.001, and ####*P* < 0.0001. [(G) and (H)] Quantification of LAMP1-positive lysosome and Vps41-HA–positive organelle position data in (A) to (F), *n* = 10 cells, respectively. (I) Quantification of Vps41-HA + LAMP1 colocalization signal data in (A) to (F), *n* = 10 cells.

Under normal conditions, the endolysosomal compartment in garland nephrocytes forms distinct layers, with the lysosomal layer partially overlapping with and situated slightly below the late endosomal layer (Fig. 8, A, G, H, and I). Immunostaining revealed that Syx17 RNAi cells mostly lack HOPS-positive endosomes, and HOPS redistributes to clusters at the periphery, where its colocalization with Lamp1 markedly increases (Fig. 8, B, H, and I). Moreover, far fewer lysosomes are present in their respective layer compared to controls (Fig. 8, B, G, and I). In contrast, Atg14 or Plekhm1 depletion yields nephrocytes resembling controls (Fig. 8, C, E, G, H, and I). Depletion of Atg14 or Plekhm1 in Syx17 RNAi cells restores the recruitment of HOPS to endosomes and normalizes lysosomal distribution (Fig. 8, D, F, G, H, and I). These findings demonstrate that the entrapment of lysosomes and the HOPS tethering complex into clusters inhibit endolysosome maturation in Syx17-depleted cells.

## DISCUSSION

The first identified metazoan autophagic SNARE complex comprises Syx17 (STX17 in mammals), Snap29, and Vamp7 (*14*, *15*). While additional autophagic SNARE complexes have been found in mammalian cells (*18*–*20*), in insects, this complex remains the sole fusogenic autophagic SNARE complex identified thus far (*15*, *21*). Syntaxin17 is a Qa-SNARE belonging to the endosomal Qa-SNARE group (*41*); however, its depletion did not cause any endocytosis defect either in cultured mammalian cells or fly fat or developing eye cells (*14*, *15*). We assumed that this is because those studies examined cells with low-average endocytic activity and that Syx17 may have endocytic functions in cells in which endocytosis proceeds at elevated rates. *Drosophila* larval garland nephrocytes exhibit continuously high endocytic activity, and we have already used this cell type to characterize previously unknown players in endolysosomal trafficking successfully (*9*, *28*, *29*).

Endolysosomal maturation is inhibited, and autophagosome-lysosome clusters appeared both in Syntaxin17-depleted fly and HEK-293 cells. Moreover, Syx17-depleted nephrocytes or salivary gland cells were unable to secrete autophagosomes. A similar finding was shown by others using *Drosophila* imaginal disc or cultured mammalian cells in which cells depleted of fly or mammalian Snap29 or VAMP8 get rid of unfused autophagosomes by secretion, unlike Syx17/STX17-depleted cells (*37*, *53*, *62*). However, the difference between Syntaxin17, Snap29, and VAMP8 has never been addressed in this context, yet.

Our results suggested at first that Syntaxin17 may have unrecognized roles: involvement in endosome maturation and autophagosome secretion. The former suggestion is supported by bioinformatics, which places Syx17 among endosomal SNAREs (*41*), while the latter is backed by its localization on autophagosomes (*14*, *15*). However, our in-depth experiments showed that neither of these hypothesized functions appear to be true. First, inhibiting autophagosome formation rescued the endolysosomal maturation defects. If Syx17 were an endosomal SNARE, inhibition of autophagosome formation should not have affected the endosomal phenotype. Second, depleting HOPS or its recruiting small GTPases (Rab7, Rab2, or Arl8) rescued autophagosome secretion in Syx17 knockdown cells. If Syx17 were genuinely required for autophagosome secretion, autophagosomes should not have been secreted in these cases.

Although Syx17 does not appear to be directly involved in autophagosome secretion or endosome maturation, it may still act as a regulatory factor in these processes. Its abundance could influence HOPS or lysosome availability for endosome maturation or autophagosome availability for secretion when lysosomal degradation is impaired. A somewhat analogous phenomenon has been observed with the CORVET and HOPS complexes. Although these two tethering complexes function independently (CORVET primarily regulating early endosomes and HOPS late endosomes and lysosomes), the availability of HOPS can still be influenced by CORVET. Specifically, in both fly and mammalian cells, high levels of the CORVET-specific subunit Vps8 were found to reduce the pool of HOPS, potentially by sequestering shared components (*28*, *63*). However, unlike Vps8, Syx17 may influence HOPS function through a different mechanism: by “unlocking” tethering lock, as further discussed later.

Secretory autophagy, an unconventional mode of secretion, is characterized by the selective sequestration of cytosolic proteins into autophagosomes that fuse with the plasma membrane (*64*, *65*). Our results, along with findings from others (*53*, *54*), suggest that this mechanism also serves as a nonselective pathway for eliminating degradatory autophagosomes when autophagosome-lysosome fusion fails. This dual role underscores the versatility of secretory autophagy in maintaining cellular integrity and highlights its potential significance in pathological conditions where conventional degradation pathways are compromised.

The exocyst is the primary tethering complex in secretory pathways and exocytosis (*56*), and it is plausible that it also facilitates autophagosome secretion, although this has not been previously demonstrated. Our findings suggest that the exocyst complex plays a role in autophagosome secretion, but its precise function remains to be fully elucidated.

Some—if not all—secreted autophagosomes are directed into the lacunar channels beneath the filtration diaphragm in fly nephrocytes. However, neither the diaphragms nor the morphology of the lacunar channels appears to be affected by these secreted autophagosomes. This could be due to their reabsorption or disintegration within the lacunar lumen, an aspect that warrants further investigation. Emerging evidence highlights autophagy as a critical factor in human kidney diseases (*66*). Since autophagosome secretion may provide a means to clear cytosolic material without compromising the filtration barrier, it could serve as an alternative pathway when lysosomal function is impaired in kidney cells.

Plekhm1 regulates lysosome positioning and homeostasis, and it contributes to autophagosome-lysosome fusion via binding to Rab7, Arl8, and HOPS (*6*, *9*, *67*–*69*). This protein is not essential in *Drosophila* (*60*). We found its depletion to cause a mild defect in autophagic but not in endosomal degradation. The observed autophagosome-lysosome clusters in Syx17-depleted cells are held together by the HOPS tethering complex, trapping this tether along with autophagosomes and lysosomes. The cluster formation can be inhibited by Plekhm1 loss, leading to release of the entrapped autophagosomes, HOPS, and lysosomes, thus rescuing all observed secondary effects of Syx17 depletion arising in addition to its primary defect in autolysosomal degradation. Our results have shed light on how HOPS and Plekhm1 may function. Plekhm1 seems to stabilize HOPS-induced tethering, perhaps without being necessary for this process. This would explain why HOPS-mediated fusion can occur in *Plekhm1* mutants (*60*), but stable HOPS-tethered vesicle clusters cannot be observed in Plekhm1-Syx17 double RNAi or mutant cells.

In recent years, studies using purified liposomes have yielded valuable insights into HOPS-mediated membrane fusion. Notably, the Wickner lab demonstrated that HOPS could recognize each SNARE involved in vacuolar fusion, leading to the assembly of ternary trans-SNARE complexes that facilitate rapid fusion upon engagement with the fourth SNARE. Through coincubation experiments with proteoliposomes bearing R-SNARE and those with any two Q-SNAREs a “rapid-fusion” complex comprising three SNAREs in a trans-assembly was observed. Adding the missing Q-SNARE then induced sudden fusion, hence the term rapid-fusion (*70*, *71*). Similarly, HOPS was found to assemble a rapid-fusion complex between R- and QbQc-SNARE containing proteoliposomes in the absence of Qa-SNARE, awaiting Qa for fusion (*70*). On the basis of these and our results, we propose the potential existence of a prolonged tethering state between vesicles in vivo under certain conditions if specific SNARE proteins are missing.

**Fig. 9.**
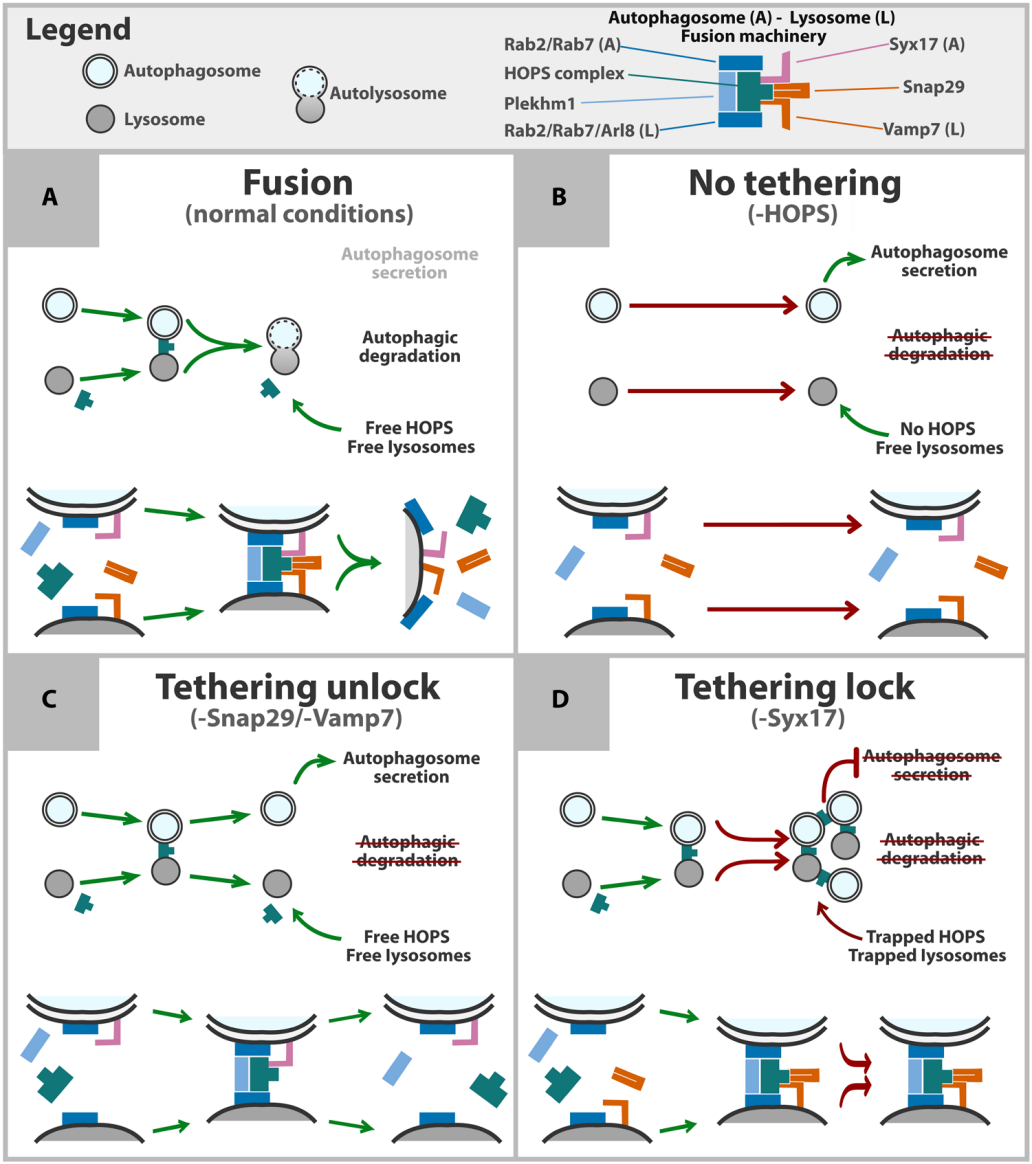
Model of tethering lock formation upon loss of Syntaxin17. The upper part of the figure illustrates how vesicles and proteins are represented in this model. In control cells (**A**), autophagic degradation is mediated by HOPS, which tethers Rab2- and Rab7-positive autophagosomal membranes to Rab2-, Rab7-, and Arl8-positive (endo)lysosomal membranes. This tethering is stabilized by Plekhm1, and fusion is executed by the Syx17-SNAP29-Vamp7 SNARE complex. In the absence of HOPS (**B**), tethering does not occur, and undegraded autophagosomes can be secreted as an alternative clearance mechanism. While lysosomes remain available, they cannot participate in HOPS-dependent trafficking routes due to the lack of tethering. When SNAP29 or Vamp7 is absent (**C**), HOPS tethers autophagosomes to lysosomes, but fusion cannot proceed. In this case, Syx17 unlocks the tethered state, freeing autophagosomes, lysosomes, and HOPS. As a result, undegraded autophagosomes are secreted, and both HOPS and lysosomes remain available for other cellular processes that do not depend on SNAP29 or Vamp7. In contrast, in the absence of Syx17 (**D**), Plekhm1-stabilized tethering creates a tethering lock, which persists independently of SNAP29 or Vamp7. This locked state traps autophagosomes, lysosomes, and HOPS, preventing their involvement in other essential processes such as endosome maturation and autophagosome secretion. Thus, Syx17 depletion indirectly affects these pathways by locking the system in a tethered state, beyond Syntaxin 17’s canonical role in autophagosome-lysosome fusion.

We suppose that the formation of autophagosome-lysosome clusters in cells depleted of Syntaxin17 is driven by this mechanism: a ternary trans-assembled SNARE complex involving Vamp7 and Snap29 may form between HOPS-tethered autophagosomes and lysosomes, awaiting Syx17. Since Syx17 is the only remaining component of the fusion machinery, the Plekhm1-stabilized HOPS-dependent tethering appears to be too stable to resolve in Syx17-depleted nephrocytes, resulting in a state what we propose to term “tethering lock.” In other words, HOPS, Plekhm1, and small GTPases together form a stable “glue” that holds together autophagosomes and lysosomes without being able to resolve in the absence of the autophagosomal SNARE Syx17. This stability results in both the tethered organelles and HOPS becoming insufficient in other cellular processes in which they would normally participate (Fig. 9).

Double RNAi of Syx17 with SNAP29 or Vamp7, along with the triple RNAi of all three SNAREs, also induced cluster formation, thus tethering lock. This phenomenon appears to occur independently of the participating SNAREs, but it is not seen whether Syx17 is present as clusters were rarely or never observed in SNAP29 or Vamp7 single RNAi cells, respectively. The mechanism by which Syx17 achieves this effect remains an interesting direction for future studies. Notably, such cluster formation has not been reported in other Syntaxin17-depleted fly fat, developing eye, or cultured mammalian cell types as, apart from autophagosome maturation, most HOPS-dependent processes remained unaffected (*14*, *15*, *27*). This suggests that these cells have an abundance of available lysosomes and HOPS that remain sufficient even when a pool of these is locked with/on autophagosomes. Despite this, autophagosome secretion remains inhibited in the absence of Syntaxin17 (*37*, *62*), indicating that lysosome tethered autophagosomes cannot be removed via secretion. We observed autophagosome-lysosome cluster formation in siSTX17 RNA-treated cultured HEK-293 cells. This phenotype was not rescued by cotransfection with an siRNA-resistant STX17 containing Ala substitutions of Lys and Arg amino acid residues near its TM domains that disrupts the autophagosomal recruitment of STX17 (*16*). This finding suggests that STX17 can resolve tethering lock only when properly localized to the autophagosomal membrane. Furthermore, a recent study in murine osteoblasts have shown that dephosphorylation of STX17 at Tyr^157^ and Tyr^227^ by protein tyrosine phosphatase 1B reduces the formation of the STX17-SNAP29-VAMP8 SNARE complex, impairing autophagosome-lysosome fusion while shifting the cells toward a secretory autophagy pathway (*55*). Notably, Tyr^157^ and Tyr^227^ have not been implicated in targeting STX17 to the autophagosomal membrane. Therefore, we propose that dephosphorylation at these residues may enable STX17 to actively inhibit or destabilize autophagosome-lysosome tethering, thereby promoting autophagosome secretion.

It is a crucial aspect of our findings that tethering lock in the absence of Syx17 disrupts cellular processes in which Syx17 is not directly involved. Since the characterization of proteins including SNAREs often relies on loss-of-function studies, the effect of tethering lock can be an important factor, causing secondary effects unrelated to the true function of a studied protein. Our experiments have shown that loss of Vamp7 or Snap29 does not induce autophagosome-lysosome tethering lock, but when Syx17 is also removed, the lock occurs. These suggest that the occurrence of tethering lock may be specific to certain SNARE-tether combinations in vivo. With several other tethering and SNARE complexes identified, it is an important future direction to investigate whether other tethering lock states arise in the absence of other SNAREs, or whether this is a unique property of Syntaxin17.

## MATERIALS AND METHODS

Reagents and tools used in this study are listed in Table 1.

**Table 1.** Reagents and tools used in this study. DMEM, Dulbecco’s modified Eagle’s medium; RIPA, radioimmunoprecipitation assay.

Reagent or resource	Source	ID
Antibodies		
Alexa Fluor 488 donkey anti-rat (dilution 1:600)	Thermo Fisher Scientific Inc.	AB_2535794
Alexa Fluor 488 goat anti-chicken (dilution 1:600)	Thermo Fisher Scientific Inc.	AB_2534096
Alexa Fluor 488 goat anti-rabbit (dilution: 1:1000)	Abcam Limited	AB_2630356
Alexa Fluor 568 donkey anti-mouse (dilution: 1:1000)	Thermo Fisher Scientific Inc.	AB_11180865
Alexa Fluor 568 donkey anti-rabbit (dilution: 1:1000)	Thermo Fisher Scientific Inc.	AB_2534017
Alexa Fluor 568 goat anti-rat (dilution: 1:1000)	Thermo Fisher Scientific Inc.	AB_2534121
Alexa Fluor 647 donkey anti-rabbit (dilution 1:600)	Thermo Fisher Scientific Inc.	AB_2536183
Anti-Atg8a (rat, dilution: 1:300)	(*15*)	–
Anti-CathL (rabbit, dilution: 1:100)	Abcam Limited	AB_940826
Anti-cubulin (rat, dilution: 1:200)	(*77*) Provided by J. Culi	
Anti-HA (rabbit, dilution 1:100)	Merck KGaA	AB_260070
Anti-HA (rat, dilution 1:100)	Roche Holding AG	AB_2314622
Anti-Lamp1 (rabbit, dilution 1:1000)	(*78*)	-
Anti-p62/Ref2p (rabbit, dilution 1:1000)	(*79*)	AB_2569199
Anti-Pyd (mouse, dilution 1:400)	Developmental Studies Hybridoma Bank	AB_2618043
Anti-Rab5 (rabbit, dilution: 1:100)	Abcam Limited	AB_882240
Anti-Rab7 (mouse, dilution: 1:10)	Developmental Studies Hybridoma Bank	AB_2722471
Anti-Snap29 (rat, dilution 1:300)	(*15*)	–
Anti-Sns (chicken, dilution 1:1000)	(*72*)	–
Anti-STX17 (rabbit, dilution 1:1000)	Sigma-Aldrich	HPA001204
Anti-tubulin(α) (mouse, dilution 1:5000)	Thermo Fisher Scientific Inc.	62204
Anti-Vps16a (rabbit, dilution: 1:50)	(*80*)	AB_2569229
IRDye 680RD goat anti-mouse	LI-COR Environmental GmbH	926-68070
IRDye 800CW goat anti-rabbit	LI-COR Environmental GmbH	925-32211
Chemicals and other reagents		
Phosphate-buffered saline (PBS)	Merck KGaA	P-3813
4′,6-Diamidino-2-phenylindole dihydrochloride (DAPI)	Merck KGaA	D8417
AgNO_3_	Merck KGaA	209139
Albumin from bovine serum (BSA) Alexa Fluor 647	Thermo Fisher Scientific Inc.	A34785
Calcium chloride	Merck KGaA	C1016
Durcupan	Merck KGaA	44614
Fetal bovine serum (FBS)	Merck KGaA	F4135
FITC-avidin	Thermo Fisher Scientific Inc.	434411
Formaldehyde solution	Merck KGaA	252549
Glutaraldehyde solution	Merck KGaA	G7776
Lead citrate	Thermo Fisher Scientific Inc.	A10701.22
Osmium tetroxide	Merck KGaA	O5500
Paraformaldehyde	Merck KGaA	P6148
Shield and Sang M3 insect medium	Merck KGaA	S8398
Sodium cacodylate trihydrate	Merck KGaA	C0250
Sucrose	Merck KGaA	S0389
Tannic acid	Mallinckrodt	126420
Triton X-100	Merck KGaA	X100
Uranyl acetate EM solution	TAAB	U001/S/2/10
VECTASHIELD antifade mounting medium	Vector Laboratories Inc.	H-1000-10
Cell line, cell culture media, and transfection reagents		
0.1% SDS	Sigma-Aldrich	151-21-3
10% Fetal bovine serum (FBS)	Euro Clone	ECS0183L
50 mM tris	Sigma-Aldrich	77-86-1
DMEM medium	Sigma-Aldrich	D6429
HBSS (Hanks’ balanced salt solution) - phenol red free	Sigma-Aldrich	55037C
HEK-293 cells	American Type Culture Collection (ATCC)	CRL-1573
LAMP1-mGFP	Addgene	34831
Lipofectamine RNAiMAX	Thermo Fisher Scientific Inc.	13778
Penicillin and streptomycin mixture	Cytiva	SV30010
pmRFP-LC3	Addgene	21075
Protease inhibitor cocktail	Sigma-Aldrich	P8340
RIPA buffer	Sigma-Aldrich	H3375
siSTX17	(*16*) Integrated DNA Technologies	–
STX17(R/K > A)	(*16*)	
STX17 (WT)	(*16*)	–
*Drosophila* stocks		
*Mutants*		
Plekhm1 [d18]	(*60*)	FBal0376288
Syx17 [LL06330]	Drosophila Genomics and Genetic Resources (DGGR), Kyoto, Japan	FBst0319577
Vps16A [d32]	(*40*)	FBal0296357
White [1118]	Bloomington Drosophila Stock Center (BDSC)	FBst0003605
*Reporters and drivers*		
3xmCherry-Atg8a	(*48*)	FBal0325100
Gen-vps8-9xHA	(*29*)	FBal0320419
Prospero-Gal4	Bloomington Drosophila Stock Center (BDSC)	FBst0080572
sns-sfGFP	(*81*) Provided by T. Hermle	FBal0376697
UAS-2xEGFP	Bloomington Drosophila Stock Center (BDSC)	FBst0006874
UAS-Arl8.GFP	(*35*) Provided by S. Munro	FBal0344485
UAS-GFP.Sec15	Bloomington Drosophila Stock Center (BDSC)	FBst0039685
UAS-GFP-myc-2xFYVE	Bloomington Drosophila Stock Center (BDSC)	FBst0042712
UAS-GFP-VAMP7	(*82*) Provided by A. Kiger	FBal0316220
UAS-Past1.B.GFP	Bloomington Drosophila Stock Center (BDSC)	FBst0081056
UAS-Rbsn-5.ORF.3xHA	Zurich ORFeome Project (FlyORF), Switzerland	FBst0501500
UAS-RFP-KDEL	Bloomington Drosophila Stock Center (BDSC)	FBst0030910
UAS-Syx17.Z.EGFP	(*83*) Provided by J. C. Pastor-Pareja	FBtp0157316
UAS-Vps41-9xHA	(*28*)	FBal0296357
*RNAis*		
UAS-Arl8 [7891R-1]	National Institute of Genetics Fly Stocks (NIG-FLY) Mishima, Japan	FBal0275763
UAS-Atg1 [GD7149]	Vienna Drosophila Resource Center (VDRC)	FBst0452169
UAS-Atg14 [KK100903]	Vienna Drosophila Resource Center (VDRC)	FBst0480369
UAS-Ccz1 [GD8501]	Vienna Drosophila Resource Center (VDRC)	FBst0453144
UAS-Def8 [11534R-3]	National Institute of Genetics Fly Stocks (NIG-FLY) Mishima, Japan	FBal0270900
UAS-Luciferase [JF01355]	Bloomington Drosophila Stock Center (BDSC)	FBst0031603
UAS-Mon1 [11926R-1]	National Institute of Genetics Fly Stocks (NIG-FLY) Mishima, Japan	FBal0271032
UAS-Past1 [HMS00557]	Bloomington Drosophila Stock Center (BDSC)	FBst0033689
UAS-Plekhm1 [GD11978]	Vienna Drosophila Resource Center (VDRC)	FBst0464428
UAS-Rab2 [GD11158]	Vienna Drosophila Resource Center (VDRC)	FBst0460794
UAS-Rab7 [GD11800]	Vienna Drosophila Resource Center (VDRC)	FBst0463506
UAS-Rab7 [JF02377]	Bloomington Drosophila Stock Center (BDSC)	FBst0027051
UAS-Rbsn5 [HMC04769]	Bloomington Drosophila Stock Center (BDSC)	FBst0057459
UAS-Sec15 [JF02649]	Bloomington Drosophila Stock Center (BDSC)	FBst0027499
UAS-Sec5 [JF02676]	Bloomington Drosophila Stock Center (BDSC)	FBst0027526
UAS-Snap29 [HMC03467]	Bloomington Drosophila Stock Center (BDSC)	FBst0051893
UAS-Snap29 [JF01883]	Bloomington Drosophila Stock Center (BDSC)	FBst0025862
UAS-Snap29 [KK108034]	Vienna Drosophila Resource Center (VDRC)	FBst0479760
UAS-Syntaxin17 [JF01937]	Bloomington Drosophila Stock Center (BDSC)	FBst0025896
UAS-Syx17 [GD14850]	Vienna Drosophila Resource Center (VDRC)	FBst0461758
UAS-Vamp7 [1599R-1]	National Institute of Genetics Fly Stocks (NIG-FLY) Mishima, Japan	FBal0272351
UAS-Vamp7 [GL01524]	Bloomington Drosophila Stock Center (BDSC)	FBst0043543
UAS-Vps11 [KK102566]	Vienna Drosophila Resource Center (VDRC)	FBst0479241
UAS-Vps16A [GD13782]	Vienna Drosophila Resource Center (VDRC)	FBst0455191
UAS-Vps18 [KK102176]	Vienna Drosophila Resource Center (VDRC)	FBst0478876
UAS-Vps33A [GD1397]	Vienna Drosophila Resource Center (VDRC)	FBst0466154
UAS-Vps33B [HMS02720]	Bloomington Drosophila Stock Center (BDSC)	FBst0044006
UAS-Vps39 [GD12152]	Vienna Drosophila Resource Center (VDRC)	FBst0463552
UAS-Vps41 [18028R-2]	National Institute of Genetics Fly Stocks (NIG-FLY) Mishima, Japan	FBal0399945
UAS-Vps45 [HMS01696]	Bloomington Drosophila Stock Center (BDSC)	FBst0038252
UAS-Vps8 [KK100319]	Vienna Drosophila Resource Center (VDRC)	FBst0477778
Software		
Adobe Photoshop CS4	Adobe Inc.	SCR_014199
GraphPad Prism	GraphPad Software Inc.	SCR_002798
ImageJ	ImageJ	SCR_003070
iTEM	Olympus Soft Imaging Solutions GmbH, Münster, Germany	–
LI-COR Image studio software	LI-COR Environmental GmbH	–
Zeiss Efficient Navigation 2 software	Carl Zeiss AG	SCR_013672

### Fly work and treatments

Flies were raised at 25°C on regular food. We used wandering L3 staged animals (both female and male) for our experiments. The genotype of the flies used in this study is summarized in table S1.

Silver-nitrate uptake experiments were performed as described (*29*). Briefly adult flies were allowed to lay eggs onto 0.005% AgNO_3_ containing food (20 g of yeast in 35 ml of 1.5% agar). Nephrocytes of the progeny were dissected in phosphate-buffered saline (PBS) and fixed with 4% formaldehyde in PBS [30 min at room temperature (RT)] and then photographed. Images were captured with an AxioCam ICc camera on an AxioImager Z1 microscope using a Plan-Neofluar 40×/0.75 numerical aperture objective. Before imaging, garland nephrocytes were exposed to ultraviolet light for 10 to 15 s using the microscope’s HBO 100 lamp and 4′,6-diamidino-2-phenylindole (DAPI) filter set (#49) to turn the yellowish hue of intracellular silver inclusions to brown.

### Immunohistochemistry

Nephrocytes were dissected in ice-cold PBS and fixed with 4% formaldehyde in PBS (for 45 min at RT). Samples were then washed three times for 10 min each at RT. Subsequently, permeabilization was performed in PBS containing 0.1% Triton X-100 (PBTX) for 15 min at RT, followed by blocking in a solution containing 5.0% fetal bovine serum (FBS) in PBTX for 30 min at RT. Next, samples were incubated with primary antibodies diluted in the blocking solution overnight at 4°C. After rinsing three times and washing three times for 15 min each in PBTX at RT, samples were incubated in blocking solution for an additional 30 min at RT. This was followed by incubation with secondary antibodies in blocking solution for 3 hours at RT. Washing steps were repeated, and lastly, samples were mounted in DAPI-containing VECTASHIELD (Vector Laboratories). All experiments were repeated on a different day, yielding similar results.

To visualize slit diaphragms, nephrocytes were heat fixed (*72*). Briefly, samples were dissected in ice-cold M3 medium (Merck) and collected in Eppendorf tubes filled with cold M3. The medium was then replaced with hot (100°C) 0.03% Triton X-100 (PBTX), and the closed Eppendorf tube was immediately placed into boiling water for 20 s. Afterward, the hot PBTX was replaced with room-temperature PBTX, and the samples were processed for immunostaining as described above.

### Channel diffusion assay

To visualize lacunar channels (*73*), dissected nephrocytes were fixed with ice-cold 4% formaldehyde (in PBS) for 5 min and then incubated in fluorescent Alexa Fluor 647–bovine serum albumin (BSA) in M3 medium (Merck) for 15 min at RT. A second round of fixation was used for 45 min to fix the tracer into the lacunae. Then, the samples were washed with PBS (Merck/Sigma) three times for 10 min. Last, the samples were either mounted in DAPI-containing VECTASHIELD (Vector Laboratories) or processed for immunostainings.

### Mammalian cell cultures, plasmids, transient transfection, and Western blots

HEK-293 (American Type Culture Collection, CRL-1573) cells were cultured in a 5% CO_2_ incubator at 37°C. For cell cultures, we used the Dulbecco’s modified Eagle’s medium (Sigma-Aldrich, D6429) supplemented with 10% FBS (Euro Clone, ECS0183L), in addition to a mixture of penicillin (10,000 IU/ml) and streptomycin (10,000 μg/ml; Cytiva, SV30010).

For the siRNA transfection, HEK-293 cells were grown up to 70% confluence in six-well plates. Then, cells were transfected two times (with a 4-day interval) with 30 pmol of siSTX17 (antisense: 5′ AATTAAGTCCGCTTCTAAGGTTTCC 3′; sense: 5′ GGAAACCTTAGAAGCGGACTTAATT3′, Integrated DNA Technologies) using Lipofectamine RNAi MAX (Invitrogen).

Three days after the final transfection, cells were replated in confocal dishes for cotransfection with siRNAi-resistant rescue—and the reporter constructs. We used pmRFP-LC3 (21075; deposited by T. Yoshimori—purchased from Addgene) and LAMP1-mGFP plasmids (34831; deposited by E. Dell’Angelica—purchased from Addgene) for transient transfection (*16*). This round of transfection was carried out at around 80% confluency using TransIT-LT1 transfection reagent (Mirus 22,084,721) with the desired plasmids following the manufacturer’s instructions using 1 to 2 μg each of the plasmid DNA. Twenty-four hours posttransfection, cells were starved for 1 hour with phenol red-free HBSS (Hanks’ balanced salt solution; Sigma-Aldrich, 55037C) and monitored by microscopy.

For Western blot experiments, HEK-293 and siSTX17 cells were cultured in a 60-mm-diameter plate in normal growth media (as described above). Cells were harvested by centrifugation (at 500*g*, for 5 min, 4°C) in PBS (Capricorn Scientific, PBS-1A) and lysed for 45 min (on ice) in 50 μl of radioimmunoprecipitation assay buffer [150 mM NaCl, 1% NP-40 (Sigma-Aldrich, H3375), 0.1% SDS (Sigma-Aldrich, 151-21-3), and 50 mM tris (Sigma-Aldrich, 77-86-1), pH 7.4] containing protease inhibitor cocktail (Sigma-Aldrich, P8340). Lysates were cleared by centrifugation (15,000*g*, 10 min at 4°C), and proteins were denatured by boiling for 10 min at 95°C with 2× Laemmli buffer. Equal protein amounts (20 μg per sample) were separated by SDS–polyacrylamide gel electrophoresis (10% gel) and analyzed by Western blotting as described (*74*). The following primary antibodies were used: rabbit polyclonal anti-human STX17 (1:1000; Sigma-Aldrich, HPA001204) and mouse monoclonal α-tubulin antibody (1:5000; Thermo Fisher Scientific, 62204). Species-specific fluorescent secondary antibodies (1:15,000; LI-COR, 925-32211 and 926-68070) were used for detection of protein bands on a LI-COR Odyssey CLx fluorescent scanner, using LI-COR Image studio software.

### Uptake and degradation assay

For the uptake and degradation assay, nephrocytes were dissected in ice-cold M3 medium (Merck) and then transferred to fluorescein isothiocyanate (FITC)–avidin (1:100) containing M3 medium at RT for 5 min to facilitate internalization of the tracer into early endosomes. Subsequently, the samples were either fixed with 4% formaldehyde (for 45 min at RT) or, before fixation, incubated (chased) in tracer-free medium for 45 min at RT to allow the tracer to be transported to lysosomes and undergo degradation. Fixed samples were then washed three times with PBS (10 min each at RT). In addition, half of the chased samples remained intact, while the other half was treated with detergent [0.1% Triton X-100 (PBTX)] overnight at 4°C followed by washing with PBS (three times for 10 min at RT). It is noteworthy that fixation prevents the FITC from being washed out from intact, functional lysosomes. However, treatment with detergent allows for the removal of FITC from lysosomes. If the peptide part of the tracer is not completely degraded, then it remains fixed inside the lysosomes regardless of detergent treatment. Last, the samples were mounted in DAPI-containing VECTASHIELD (Vector Laboratories).

### Fluorescent imaging

Fluorescent images of nephrocytes were obtained at RT with an AxioImager.M2 microscope (Carl Zeiss) equipped with an ApoTome2 grid confocal unit (Carl Zeiss), using a Plan-Apochromat 63×/1.40 Oil objective (Carl Zeiss), an Orca Flash 4.0 LT scientific Complementary Metal–Oxide–Semiconductor (sCMOS) camera (Hamamatsu), and Zeiss Efficient Navigation 2 software (Carl Zeiss). Cultured HEK-293 cells were imaged on a Zeiss Axio Observer.Z1 LSM800 confocal microscope using a Plan-Apochromat 63×/1.40 oil differential interference contrast M27 objective and appropriate excitation/emission settings for enhanced green fluorescent protein and RFP. The microscope and imaging settings were identical for all experiments of the same kind. Primary images of *Drosophila* experiments were processed in Zeiss Efficient Navigation 2 (Carl Zeiss), and HEK-293 primary images were processed with the Zeiss Zen Blue software. Photoshop CS4 (Adobe) was used to produce the final figures.

### Electron microscopy

Dissected nephrocytes and salivary glands were fixed in a solution containing 3.2% paraformaldehyde and 1% glutaraldehyde, 1% sucrose, and 0.028% CaCl_2_ in 0.1 N sodium cacodylate (pH 7.4) overnight at 4°C. Samples were then postfixed in 0.5% osmium tetroxide for 1 hour and in half-saturated aqueous uranyl acetate for 30 min at RT, dehydrated in a graded series of ethanol, and embedded in Durcupan (Fluka) following the manufacturer’s recommendations. Seventy-nanometer sections were stained with Reynold’s lead citrate and viewed on a JEM-1011 transmission electron microscope (JEOL) equipped with a Morada digital camera (Olympus) using iTEM software (Olympus) at an accelerating voltage of 80 kV. As tannic acid (TA) treatment was reported to enhance the visibility of autophagic membranes (*75*), some aldehyde-fixed and OsO_4_-treated Syx17 RNAi nephrocytes were treated with 1% TA in water, then dehydrated, and embedded similarly to non–TA-treated samples.

### Quantification and statistical analysis

Fluorescent structures from original, unmodified single focal planes were quantified using ImageJ software. The signal threshold for the relevant fluorescent channel was consistently set by the same person. Cells were randomly selected for counting, and only those with nuclei in the focal plane were included to ensure that both perinuclear and peripheral regions were analyzed. Each experiment was repeated at least twice on different days.

To quantify Atg8a and p62 signals in nephrocytes, we measured the area fraction of each cell covered by the given fluorescent signal using standardized threshold settings. For each genotype, four to six animals were used, and fluorescence was measured in 20 cells total (*n* = 20). When measuring Atg8a coverage within lacunae, the channel diffusion signal was used to exclude autophagosomes outside the lacunae lumen. This was achieved using the Image Calculator function in ImageJ after appropriate thresholding.

To quantify the size of Rab7- or FYVE-GFP–positive endosomes, we measured the area of individual vesicles. Endosomes were separated using the Watershed function in ImageJ, supplemented with manual segmentation when necessary. For each genotype, four to six animals were analyzed, with endosome size measurements obtained from 10 cells, yielding a dataset of *n* = 174 to 482.

To quantify KDEL-RFP signal in nephrocytes, we measured the area fraction of each cell covered by the fluorescent signal using uniform threshold settings. For each genotype, four to six animals were used, and fluorescence was measured in 20 cells (*n* = 20).

To determine the average depth of lacunae (BSA–Alexa Fluor 647 or α-cubulin signal), we outlined the cell and measured its area. The deepest point of each lacuna determined another area, which was also measured. The difference in radius between the corresponding circles was used to approximate the average depth. Measurements were taken from 20 cells (four to five animals per genotype, *n* = 20).

To assess the enrichment of fluorescent reporters and immunostained proteins in clusters, we used a method similar to that of Zaffagnini and colleagues (*76*). The ratio of signal intensity originating from clusters to that from the cytoplasm was measured and compared to Syx17 RNAi cells expressing cytosolic enhanced green fluorescent protein (shown in fig. S3B). Using ImageJ, we overexposed the Atg8a channel to approximate cluster boundaries. The average gray value of the unaltered signal was then measured within the cluster and compared to an adjacent cytoplasmic region of equal size and shape. The cluster-to-cytoplasm ratio was calculated for each cluster. For each genotype and staining type, three to five animals were analyzed, with three clusters per cell measured across 10 cells, resulting in data from 30 clusters (*n* = 30).

In the uptake and degradation assay, fluorescence intensity (mean gray value) was quantified in 12 cells (four to six animals). To assess Vps41-HA and LAMP1 colocalization in 10 cells (four to six animals), we used the Coloc2 function in ImageJ. The peripheral region of Vps41-HA–expressing LAMP1-stained cells was determined using the Enlarge function in ImageJ. The cell outline was manually drawn, and the boundary radius was reduced by 1.5 μm.

To quantify LC3B^+^ vesicle clustering in HEK-293 cells, we measured the average distance between randomly selected LC3B^+^ vesicles and their five nearest neighbors. This measurement was taken for 10 vesicles per cell in 10 cells per treatment (*n* = 100).

To quantify STX17 protein levels in Western blots, STX17 band intensities were measured and normalized to α-tubulin staining. This experiment was repeated six times (*n* = 6).

Quantified data were analyzed using GraphPad Prism. The Gaussian distribution of datasets was assessed using the D’Agostino and Pearson normality test. For datasets with a non-Gaussian distribution, the Mann-Whitney test or Kruskal-Wallis test with Dunnett’s or Dunn’s multiple comparisons test was applied. For normally distributed datasets, the Student’s *t* test or analysis of variance (ANOVA) with an appropriate post hoc test was used. *P* value ranges were indicated as follows: or # (*P* < 0.05), ** or ## (*P* < 0.01), *** or ### (*P* < 0.001), and **** or #### (*P* < 0.0001). Figure legends specify the reference genotype for comparisons. Boxplots display minimum and maximum values, the 25th and 75th percentiles, and the mean. The median is marked by a horizontal black line within the box, with whiskers representing the smallest and largest observations. Individual data points are also shown. Details and statistical analysis results are provided in table S2.
