# Supplementary material for: HOPS-dependent vesicle tethering lock inhibits endolysosomal fusions and autophagosome secretion upon the loss of Syntaxin17
Source: Sci Adv. 2025 Jun 6;11(23):eadu9605. doi: 10.1126/sciadv.adu9605 (PMC12143387; doi:10.1126/sciadv.adu9605)
Supplement: Supplementary file 1 — Figs. S1 to S9 Tables S1 and S2 [file sciadv.adu9605_sm.pdf]

Science Advances
AAAS

Supplementary Materials for

HOPS-dependent vesicle tethering lock inhibits endolysosomal fusions and autophagosome secretion upon the loss of Syntaxin17

Dávid Hargitai *et al.*

Corresponding author: Péter Lőrincz, peter.lorincz@ttk.elte.hu

Sci. Adv. **11**, eadu9605 (2025)
DOI: 10.1126/sciadv.adu9605

This PDF file includes:

Figs. S1 to S9
Tables S1 and S2

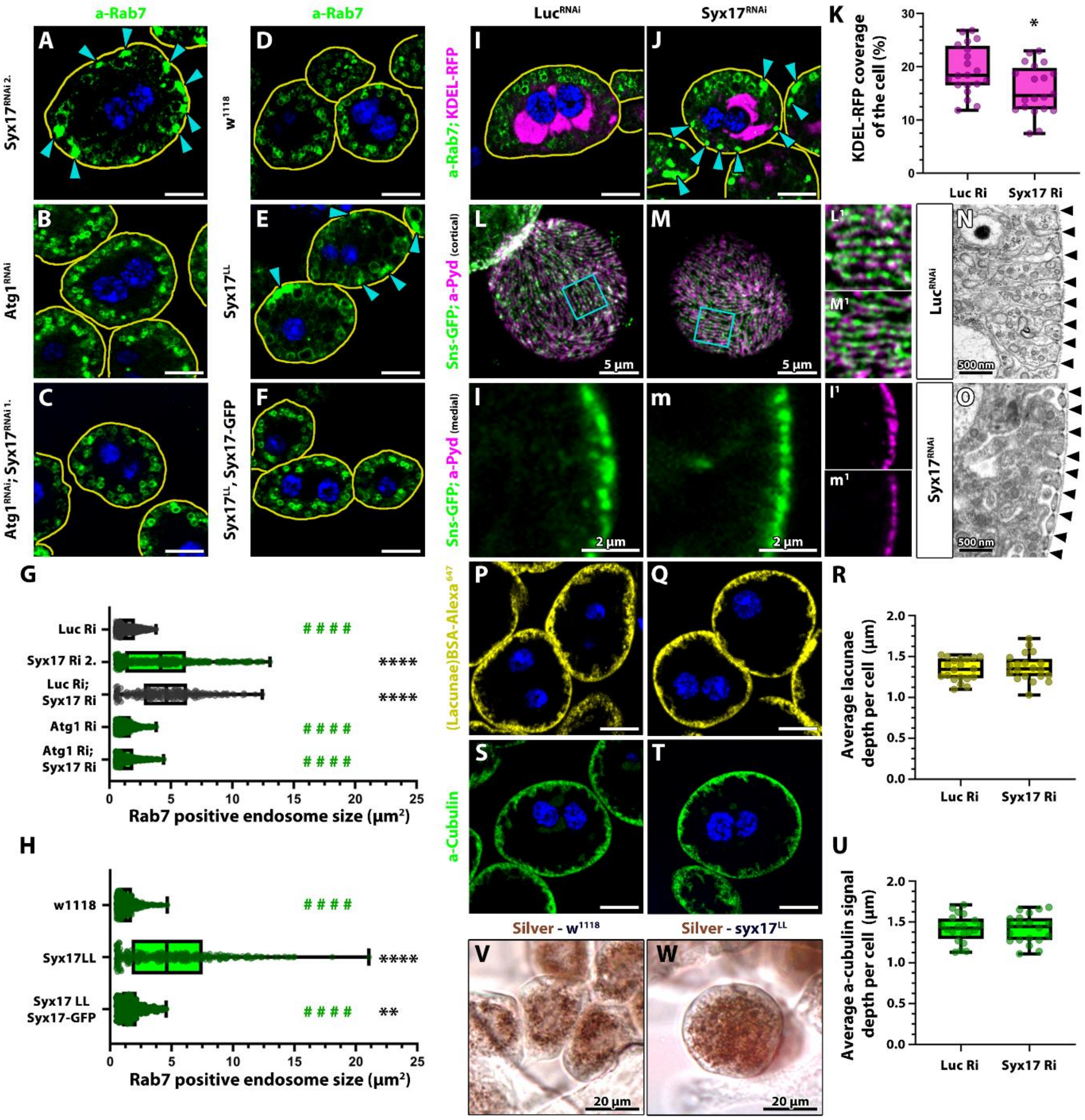

Fig. S1. Additional Syx17 loss-of function data.

(A-F) Rab7 positive late endosomes are also enlarged in Syx17 RNAi 2. or mutant nephrocytes (A, E) compared to control (D and Figure 1A) cells. Co-expressing Atg1 RNAi with Syx17 RNAi 1. restores Rab7+ endosome size (B, C). Expression of Syx17-GFP restores endosome size in *syx17* mutant cells (F). Cyan arrows on panels A, E point to Rab7 positive aggregates. Blue channel shows the nuclei of the cells (stained by DAPI). The outline of the cells is indicated by yellow lines. Scale bars: 10 µm. (G-H): Quantifications of data shown in A-F. Asterisks (*) indicate comparisons to the control, while green hashes (#) indicate comparisons to Luc RNAi; Syx17 RNAi 1. or mutant cells. **: $p<0.01$, ****: $p<0.0001$, #: $p<0.05$, ####: $p<0.0001$ (G): Quantification of Rab7 positive endosome size in A–C (shown in green) and Fig 1. A, B (shown in gray). n = 173 – 474 endosomes from 10-10 cells. (H): Quantification of Rab7 positive endosome size in D-F. n = 250 – 367 endosomes from 10-10 cells. (I, J) The endoplasmic reticulum

(ER), marked by KDEL-RFP, occupies a smaller area in *Syx17* RNAi nephrocytes (J) compared to controls (I), possibly due to Rab7-positive endosomes expanding into its space. (K) Quantification of KDEL-RFP coverage in (I, J). n = 20 cells. *: $p < 0.05$. (L-O) Slit diaphragm morphology appears similar in Syx17 RNAi nephrocytes and controls, as shown by Sns-GFP diaphragm reporter expression and anti-Pyd staining (L, l, M, m), as well as ultrastructural analysis (N, O black arrowheads point to slit diaphragms). Cortical sections (L, M) show diaphragm patterns at the cell surface and L^1, M^1 show higher magnifications of the areas in cyan rectangles. Medial sections (l, m for Sns-GFP and l^1, m^1 for Pyd) show deeper regions of nephrocytes. (P-U) Channel diffusion assays using BSA-Alexa647 (P, Q) or anti-Cubulin staining (S, T) show no difference in lacunar channel depth along the perimeter of Syx17 RNAi and control cells, suggesting that Syx17 does not affect the recycling pathway. Scale bars: 10 μm. (R, U) Quantification of lacunar channel depth from (P, Q) and (S, T), respectively. n = 20 cells. (V, W) Silver nitrate uptake proceeds normally in both control and Syx17 mutant nephrocytes, as indicated by the presence of brownish silver inclusions in the cytosol.

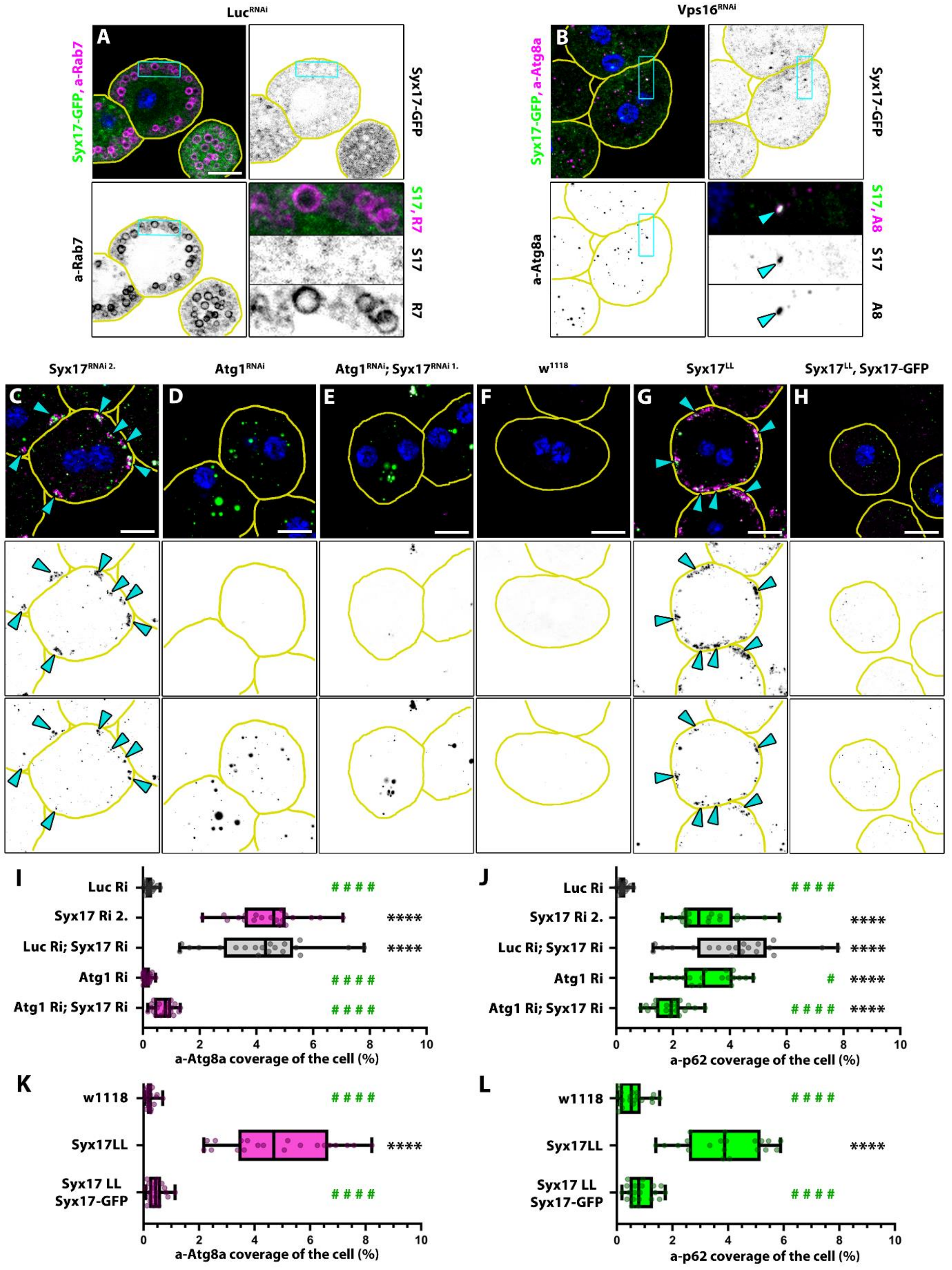

Luc^RNAi
Vps16^RNAi
A
Syx17-GFP, a-Rab7
Syx17-GFP
a-Rab7
S17, R7
S17
R7
B
Syx17-GFP, a-Atg8a
Syx17-GFP
a-Atg8a
S17, A8
S17
A8
Syx17^RNAi 2.
Atg1^RNAi
Atg1^RNAi; Syx17^RNAi 1.
w^1118
Syx17^LL
Syx17^LL, Syx17-GFP
C
D
E
F
G
H
a-p62, a-Atg8a
a-Atg8a
a-p62
I
Luc Ri
Syx17 Ri 2.
Luc Ri; Syx17 Ri
Atg1 Ri
Atg1 Ri; Syx17 Ri
####
****
a-Atg8a coverage of the cell (%)
J
#
a-p62 coverage of the cell (%)
K
w1118
Syx17LL
Syx17 LL
Syx17-GFP
L
0
2
4
6
8
10

Fig. S2. Additional Syx17 colocalization and loss-of function data.

(A, B) Syx17-GFP shows no overlap with late endosomal Rab7 (A) but colocalizes with Atg8a-positive autophagosomes (marked by cyan arrowhead) (B). It's worth noting that autophagosomes are rarely present in nephrocytes; therefore, in the latter case, we also expressed Vps16A RNAi to increase their abundance. Scale bars: 10 µm. (C-H) Atg8a/p62 positive aggregates (cyan arrows) can be detected at the cell periphery of Syx17 RNAi 2. (C) or mutant (G) nephrocytes compared to controls (F and Fig1 K) or Atg1 RNAi (D) or Atg1-Syx17 double RNAi (E) or rescued (H) cells. Of note compared to controls more p62 positive puncta can be observed in Atg1 or Atg14-Syx17 double RNAi cells, indicating effective autophagosome formation inhibition. Blue channel shows the nuclei of the cells (stained by DAPI). The outline of the cells is indicated by yellow lines. Scale bars: 10 µm. (I-L) Quantifications of data shown in C-H. Asterisks (*) indicate comparisons to the control, while green hashes (#) indicate comparisons to Luc RNAi; Syx17 RNAi or mutant cells. ****: $p<0.0001$, #: $p<0.05$, ####: $p<0.0001$, n= 20 cells in all cases. (I, J): Quantifications of Atg8a and p62 coverage in C-E (shown in magenta and green, respectively) and Fig 1. K, L (shown in gray), respectively, n = 20 cells. (K, L): Quantifications of Atg8a and p62 coverage in F-H.

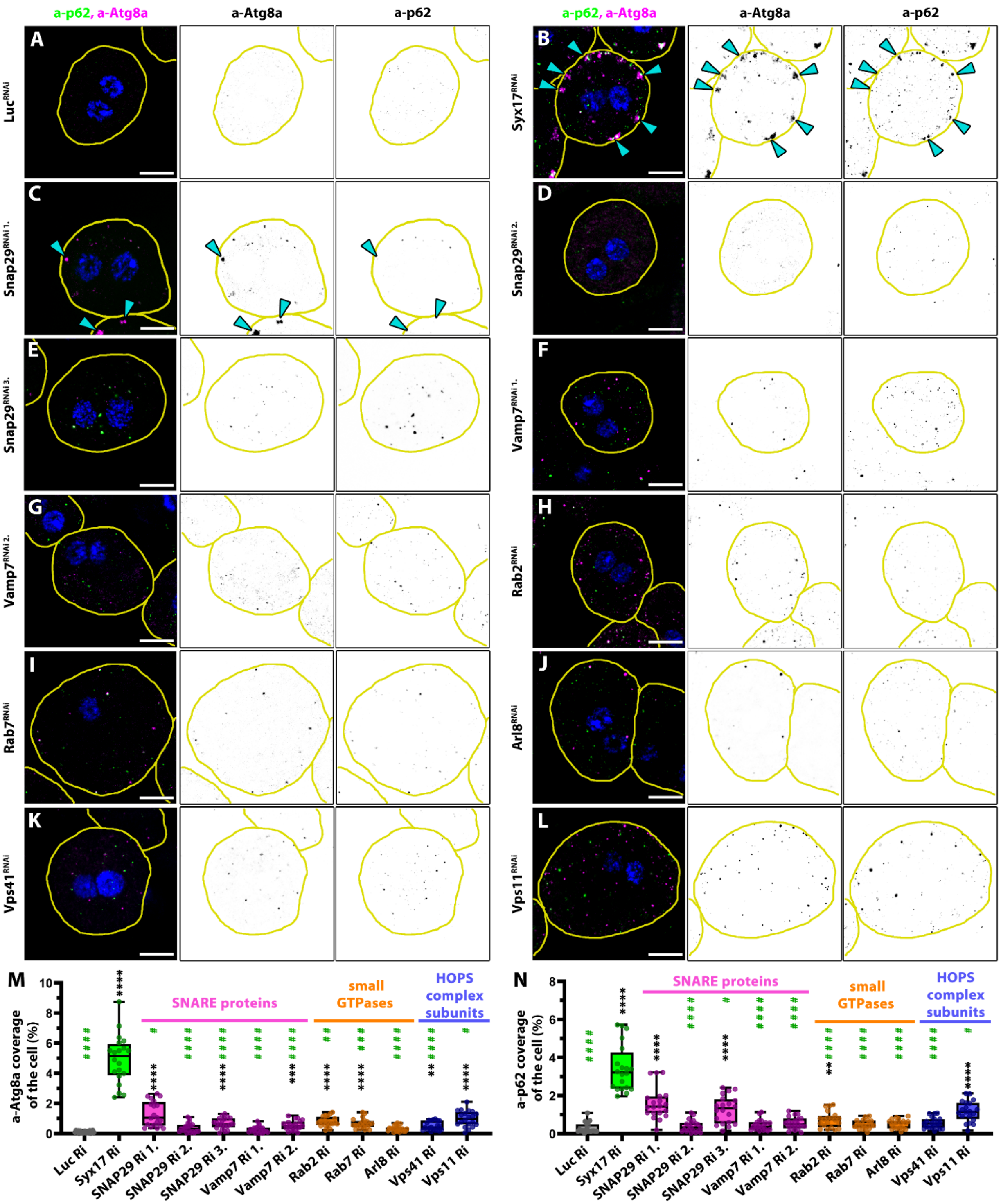

a-p62, a-Atg8a
a-Atg8a
a-p62
A
B
C
D
E
F
G
H
I
J
K
L
M
N
Luc^RNAi
Syx17^RNAi
Snap29^RNAi 1.
Snap29^RNAi 2.
Snap29^RNAi 3.
Vamp7^RNAi 1.
Vamp7^RNAi 2.
Rab2^RNAi
Rab7^RNAi
Arl8^RNAi
Vps41^RNAi
Vps11^RNAi
a-Atg8a coverage of the cell (%)
a-p62 coverage of the cell (%)
SNARE proteins
small GTPases
HOPS complex subunits
Luc Ri
Syx17 Ri
SNAP29 Ri 1.
SNAP29 Ri 2.
SNAP29 Ri 3.
Vamp7 Ri 1.
Vamp7 Ri 2.
Rab2 Ri
Rab7 Ri
Arl8 Ri
Vps41 Ri
Vps11 Ri

Fig. S3. Autophagosome clusters are rarely seen upon loss of other autophagosome-lysosome fusion factors.

(A-I) The Atg8a and p62 coverage is highest in Syx17 RNAi cells (B) compared to controls (A) or other autophagosome-lysosome fusion impaired RNAi cells, such as SNAP29 RNAi-s (C-E), Vamp7 RNAi (F, G), Rab2 RNAi (H), Rab7 RNAi (I), Arl8 RNAi (J), Vps41 RNAi (K), and Vps11 RNAi (L). Compared to Syx17 RNAi cells, in which these clusters were common (cyan arrowheads), these structures could only be rarely seen in one of the SNAP29 RNAi cells (cyan arrowheads, C) used, while they were never seen in other RNAi lines. Blue channel shows the nuclei of the cells (stained by DAPI). The outline of the cells is indicated by yellow lines. Scale bars: 10 µm. (M, N) Quantifications of data in A–I, $n = 20$ cells. Asterisks (*) indicate comparisons to the control, while green hashes (#) indicate comparisons to Syx17 RNAi cells. *: $p<0.05$, **: $p<0.01$, ***: $p<0.001$, ****: $p<0.0001$, ####: $p<0.0001$ (M) Quantification of Atg8a coverage data. Every RNAi cell had significantly lower Atg8a coverage than Syx17 RNAi. All RNAi cells, except SNAP29 RNAi 2., VAMP7 RNAi 1. and Arl8 RNAi had a higher Atg8a coverage than the control (Luciferase RNAi). (N) Quantification of p62 coverage data. Every RNAi cell had significantly lower p62 coverage than Syx17 RNAi (green #, except for a single SNAP29 RNAi) suggesting that these cells can somehow get rid of p62 positive cargo.

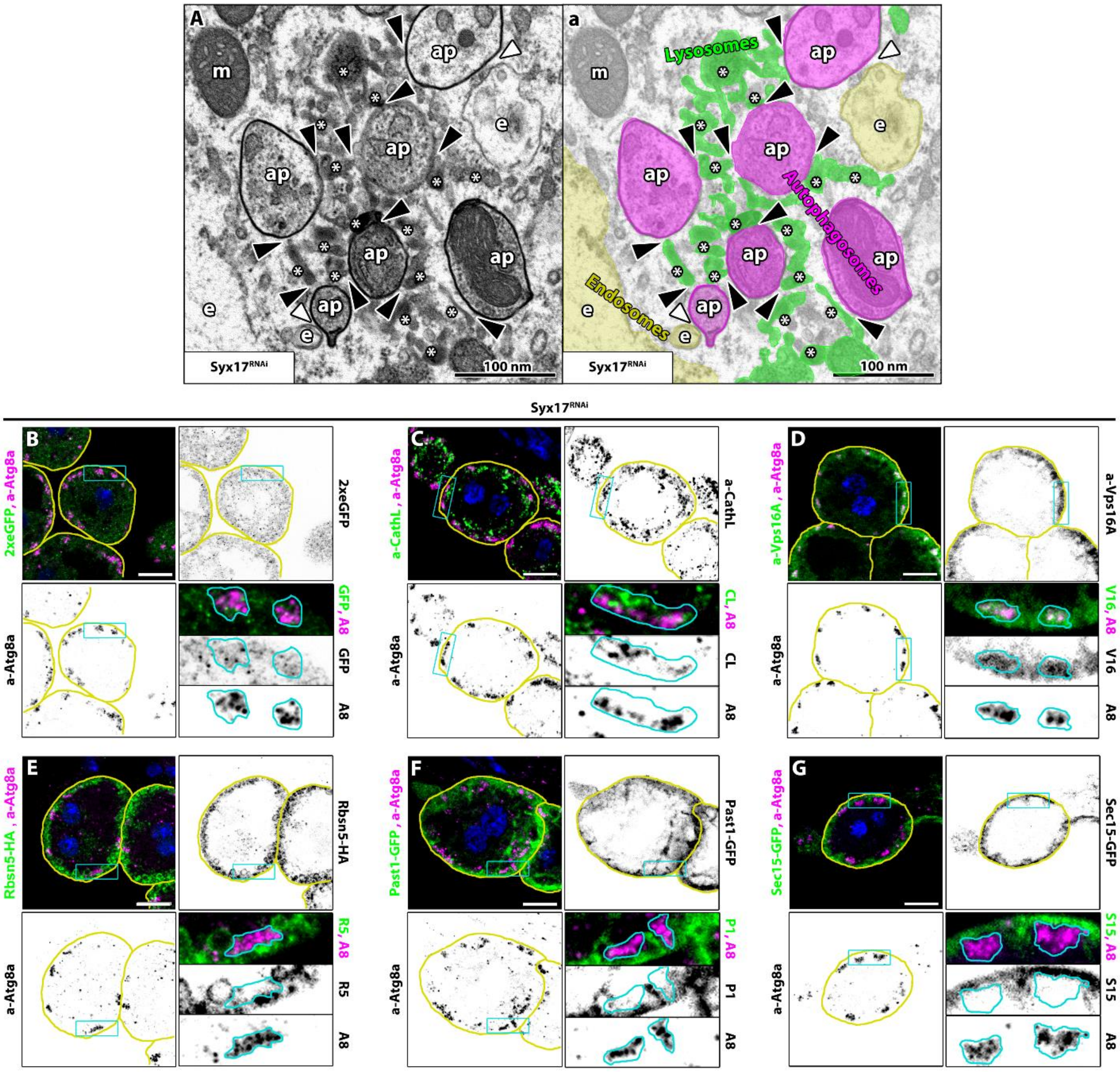
A
a
m
ap
e
Lysosomes
Autophagosomes
Endosomes
Syx17RNAi
100 nm
B
2xeGFP, a-Atg8a
2xeGFP
a-Atg8a
GFP, A8
GFP
A8
C
a-CathL, a-Atg8a
a-CathL
CL, A8
CL
D
a-Vps16A, a-Atg8a
a-Vps16A
V16, A8
V16
E
Rbsn5-HA, a-Atg8a
Rbsn5-HA
R5, A8
R5
F
Past1-GFP, a-Atg8a
Past1-GFP
P1, A8
P1
G
Sec15-GFP, a-Atg8a
Sec15-GFP
S15, A8
S15

Fig. S4. Additional data demonstrating the compartments and tethers found in the clusters of Syx17 RNAi cells.

(A, a) Additional representative electron micrograph of a cluster in a Syx17-depleted cell: fixed nephrocytes were treated with tannic acid and processed for electron microscopy to enhance the visibility of autophagosomes (ap). Lysosomes (asterisks) and some late endosomes (e) were found in the close vicinity of autophagosomes. White and black arrowheads point to endosome-autophagosome and lysosome-autophagosome contacts, respectively. (m: mitochondria). (a) False-color overlay was added to panel A to help visualization of organelles and contacts. (B) Cytosolic GFP does not accumulate in the clusters observed in Syx17 RNAi cells. (B-G) Blue channel shows the nuclei of the cells (stained by DAPI). The outline of the cells is indicated by yellow lines. Enlarged regions are marked with cyan outline as well as the approximate boundary of the enlarged clusters. Scale bars: 10 μm. (C) Fluorescent imaging revealed Cathepsin L positive structures, likely lysosomes, within the clusters of Syx17-depleted cells, exhibiting minimal overlap with Atg8a-positive autophagosomes. (D-G) Endogenous Vps16A signal was observed in the clusters (D), in contrast to Rabenosyn-5-HA (E), Past1-GFP (Ferrari subunit, F) or the Exocyst reporter Sec15-GFP (G). It's worth noting that Vps16A, being a shared subunit of both miniCORVET and HOPS complexes, could also be detected outside of the clusters, within the layer of early endosomes (D).

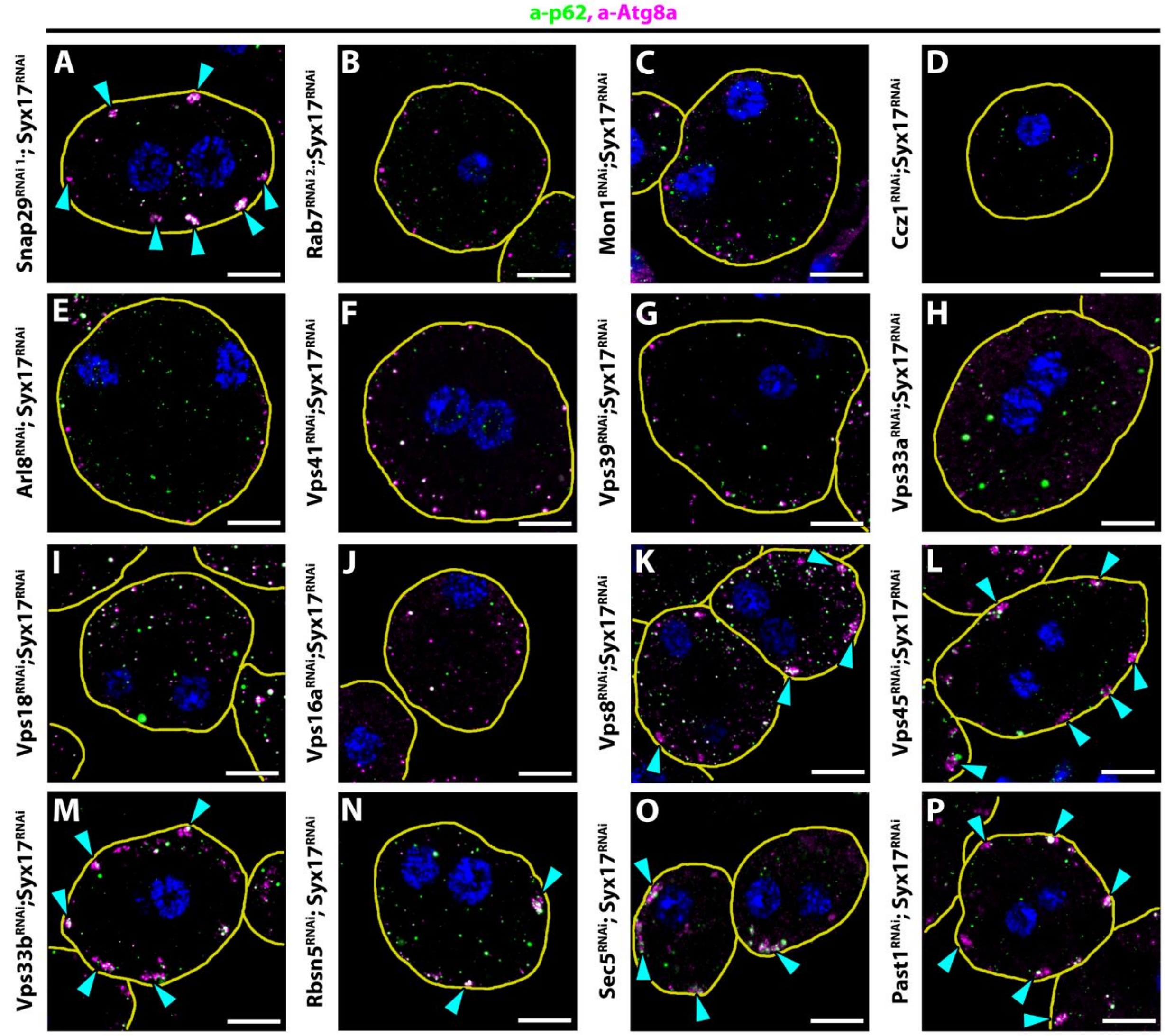

Fig. S5. Additional Atg8a and p62 data from double RNAi experiments.

(A-J) In contrast to SNAP29 1. RNAi (A) depletion of Rab7 (B), and its guanine nucleotide exchange factors (Mon1 (C), Ccz1 (D)) or Arl8 (E) or the HOPS subunits Vps41 (F), Vps39 (G), Vps33a (H), Vps18 (I) or Vps16a (J) eliminates the autophagosome clusters from Syx17 RNAi cells. Cyan arrowheads point to clusters in panel A. Blue channel shows the nuclei of the cells (stained by DAPI). The outline of the cells is indicated by yellow lines. Scale bars: 10 µm. (K-P) Depletion of other tethering factors or tethering complex subunits (Vps8 (K), Vps45 (L), Vps33b (M), Rbsn5 (N), Sec5 (O) or Past1 (P) could not eliminate the autophagosome clusters from Syx17 RNAi cells. Cyan arrowheads point to clusters in panels K-P. Blue channel shows the nuclei of the cells (stained by DAPI). The outline of the cells is indicated by yellow lines. Scale bars: 10 µm.

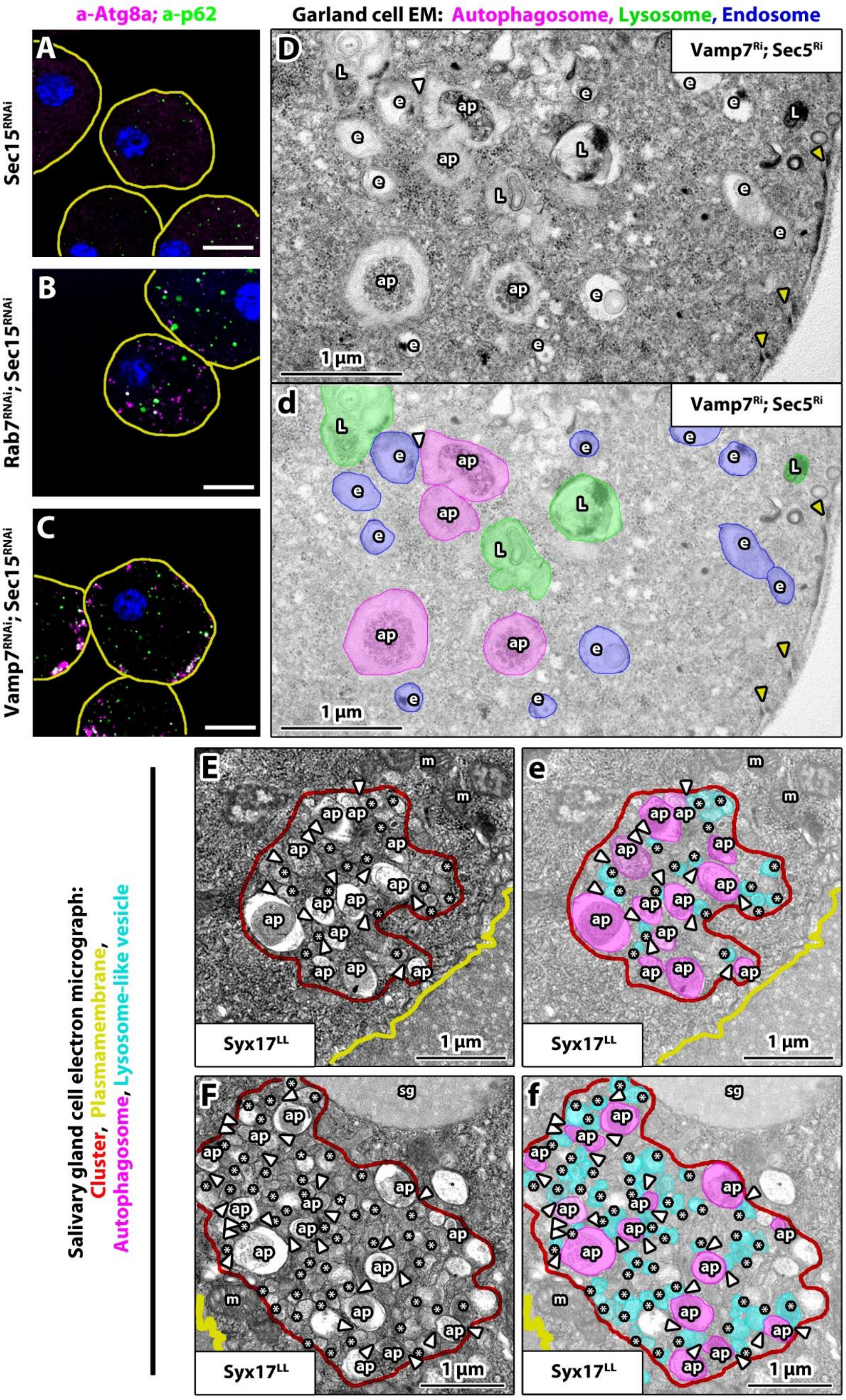
a-Atg8a; a-p62
Garland cell EM: Autophagosome, Lysosome, Endosome
A
Sec15RNAi
B
Rab7RNAi; Sec15RNAi
C
Vamp7RNAi; Sec15RNAi
D
Vamp7Ri; Sec5Ri
d
Vamp7Ri; Sec5Ri
1 µm
E
e
F
f
Syx17LL
1 µm
Salivary gland cell electron micrograph:
Cluster, Plasmamembrane,
Autophagosome, Lysosome-like vesicle

Fig. S6. Additional Exocyst data and additional salivary gland ultrastructure data.

(A-C) Increased numbers of Atg8a- and p62-positive puncta are observed in Rab7-Sec15 and Vamp7-Sec15 double RNAi nephrocytes (B, C, respectively) compared to Rab7-Luciferase or Vamp7-Luciferase double RNAi controls (shown in Figure 5A, B, respectively) or Sec15 single RNAi nephrocytes (A). (D) Autophagosome-lysosome clusters do not form in Vamp7-Sec5 double RNAi nephrocytes, as individual autophagosomes (ap) and lysosomes (L) are observed in the cytosol, in contrast to Syx17 RNAi cells (shown in Figure 2A and Figure S4G). e: endosomes. Yellow arrowheads indicate lacunar openings sealed by slit diaphragms. (E, F) Additional representative electron micrographs of clusters (outlined by red lines) in *Syx17^{LL}* mutant salivary gland cells: autophagosomes (ap), lysosome-like organelles (asterisks) White arrowheads point lysosome-autophagosome contacts, m: mitochondria, sg: secretion granule. (d, e, f) False-color overlay aids visualization of organelles and contacts. The yellow line represents the luminal plasma membrane. (E) The cell containing this cluster is shown at lower magnification in Figure 5I.

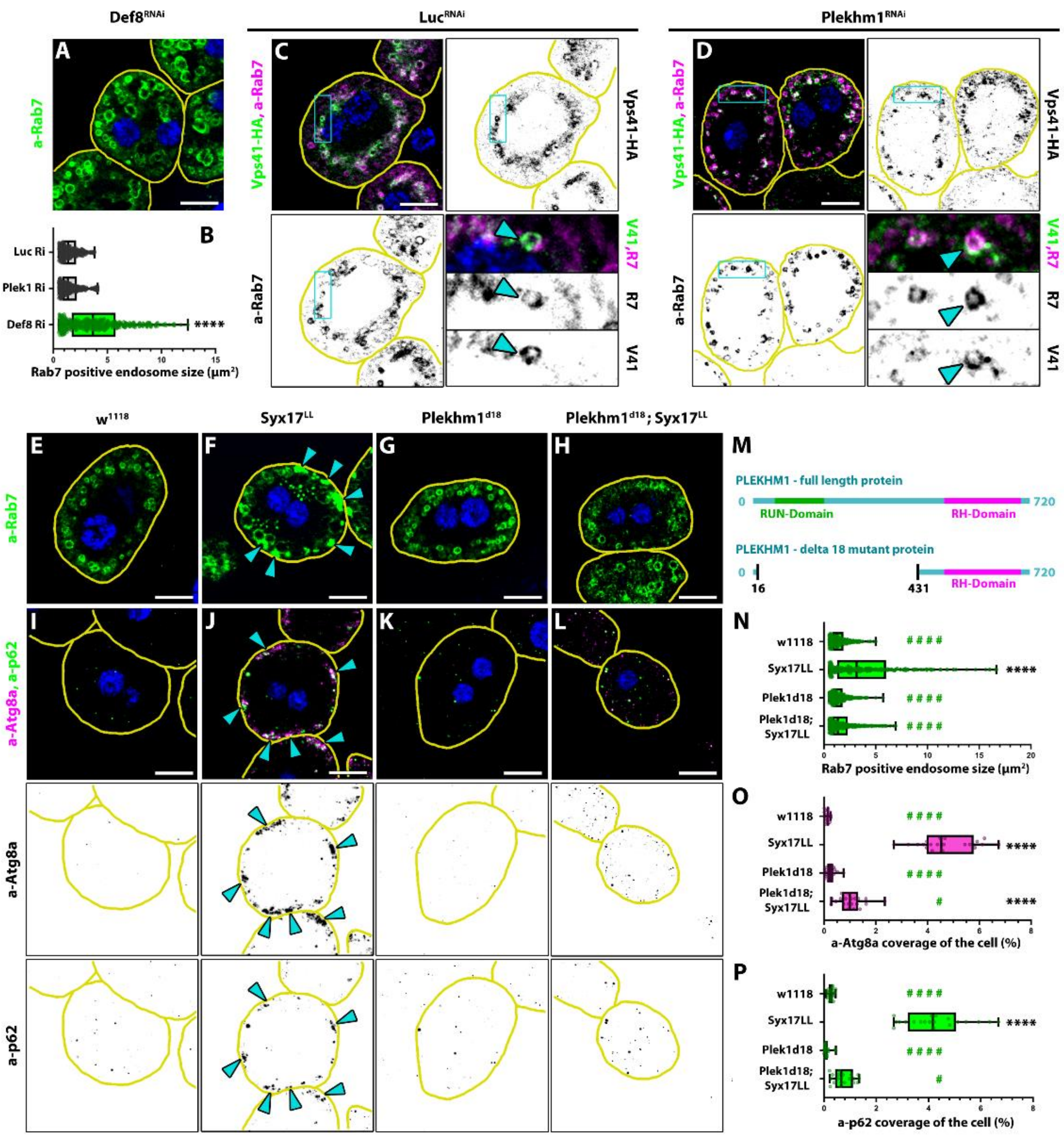

Def8RNAi
LucRNAi
Plekhm1RNAi
A
a-Rab7
B
Luc Ri
Plek1 Ri
Def8 Ri
****
Rab7 positive endosome size (µm²)
C
Vps41-HA, a-Rab7
Vps41-HA
a-Rab7
V41,R7
R7
V41
D
w1118
Syx17LL
Plekhm1d18
Plekhm1d18; Syx17LL
E
F
G
H
I
J
K
L
a-Atg8a, a-p62
a-Atg8a
a-p62
M
PLEKHM1 - full length protein
0
720
RUN-Domain
RH-Domain
PLEKHM1 - delta 18 mutant protein
16
431
N
w1118
Syx17LL
Plek1d18
Plek1d18; Syx17LL
####
#
Rab7 positive endosome size (µm²)
O
a-Atg8a coverage of the cell (%)
P
a-p62 coverage of the cell (%)

Fig. S7. Additional Plekhm1 and Def8 data.

(A) Rab7 positive late endosomes are enlarged in Def8 RNAi nephrocytes compared to control or Plekhm1 RNAi cells (Figure 5 A, B). Blue channel shows the nuclei of the cells (stained by DAPI). The outline of the cells is indicated by yellow lines. Scale bars: 10 µm. (B) Quantification of Rab7 positive endosome size data in A (shown in green) and in Figure 5 A, B (shown in gray). n = 278 – 474 endosomes from 10-10 cells. Asterisks (*) indicate comparisons to control RNAi cells. ****: p<0.0001. (C-D) Vps41-9xHA is recruited to a subset of Rab7 positive endosomes (cyan arrowheads) both in control (C) and Plekhm1 RNAi (D) nephrocytes. Of note, Rab7 positive endosome size appear similar in both cases. Blue channel shows the nuclei of the cells (stained by DAPI). The outline of the cells is indicated by yellow lines. Scale bars: 10 µm. (E-H) Rab7 positive late endosome size is similar to controls (E) in *Plekhm1*^{d18} mutant nephrocytes (G). In *Plekhm1*^{d18}*; Syx17*^{LL} double mutant cells (H), endosome size is restored compared to Syx17LL single mutants (F). Blue channel shows the nuclei of the cells (stained by DAPI). The outline of the cells is indicated by yellow lines. Cyan arrows in panel F point to Rab7 positive aggregates. Scale bars: 10 µm. (I-L) Atg8a/p62 positive clusters cannot be observed in wild type, *Plekhm1*^{d18} single or *Plekhm1*^{d18}*; Syx17*^{LL} double mutant cells (I, K, L) in contrast to *Syx17*^{LL} single mutants (J). Blue channel shows the nuclei of the cells (stained by DAPI). The outline of the cells is indicated by yellow lines. Cyan arrows in panel J point to Atg8a positive autophagosome clusters. Scale bars: 10 µm. (M) The domain organization of Drosophila Plekhm1. The [d18] deletion is an in-frame deletion removing amino acids between positions 16 and 431, including the RUN domain (N) Quantification of Rab7 positive endosome size in E–H, n = 278 – 474 endosomes from 10-10 cells. Asterisks (*) indicate comparisons to the control, while green hashes (#) indicate comparisons to *Syx17*^{LL}. ****: p<0.0001, ####: p<0.0001. (O-P): Quantifications of Atg8a and p62 coverage in K-N. Asterisks (*) indicate comparisons to the wild-type control, while green hashes (#) indicate comparisons to *Syx17*^{LL}. ****: p<0.0001, ####: p<0.0001.

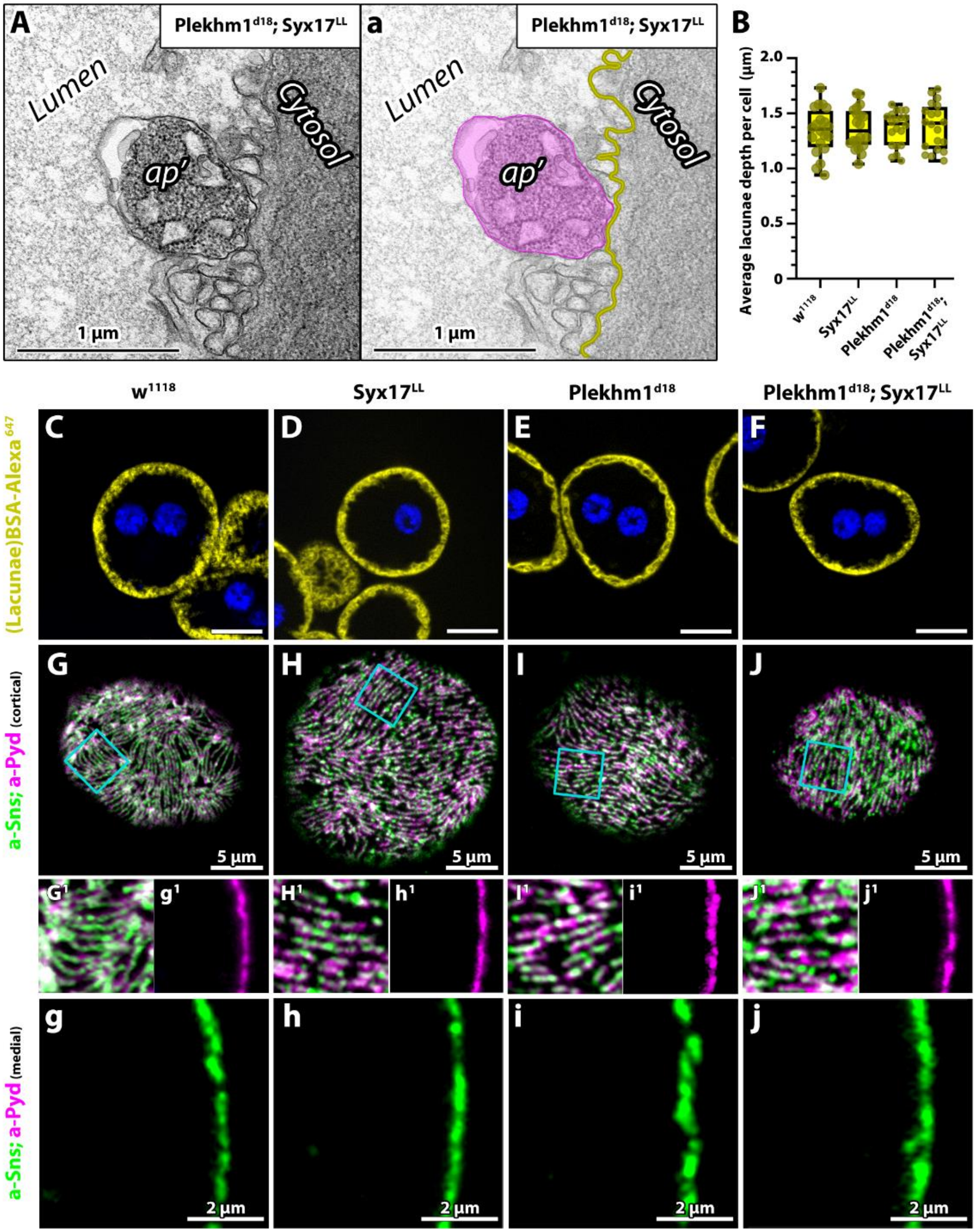

A
Plekhm1d18; Syx17LL
Lumen
Cytosol
ap'
1 µm
a
Plekhm1d18; Syx17LL
Lumen
Cytosol
ap'
1 µm
B
Average lacunae depth per cell (µm)
2.0
1.5
1.0
0.5
0
w1118
Syx17LL
Plekhm1d18
Plekhm1d18; Syx17LL
w1118
Syx17LL
Plekhm1d18
Plekhm1d18; Syx17LL
(Lacunae)BSA-Alexa647
C
D
E
F
a-Sns; a-Pyd (cortical)
G
H
I
J
5 µm
5 µm
5 µm
5 µm
G1
g1
H1
h1
I1
i1
J1
j1
a-Sns; a-Pyd (medial)
g
h
i
j
2 µm
2 µm
2 µm
2 µm

Fig. S8. Additional Plekhm1 and Syx17 mutant data.

(A) Electron microscopy revealed individual secreted autophagosomes in the salivary gland lumen of *Plekhm1^{d18}; Syx17^{LL}* double mutants. ap: autophagosome, ap': secreted autophagosome, m: mitochondria, sg: secretion granule. (a) False-color overlay aids visualization of organelles. The yellow line represents the luminal plasma membrane. (B-F) Channel diffusion assays using BSA-Alexa647 show no difference in lacunar channel depth along the perimeter of wild type controls (C), *Syx17^{LL}* and *Plekhm1^{d18L}* single (D, E, respectively) and *Plekhm1^{d18}; Syx17^{LL}* double mutants (F). Scale bars: 10 µm. (B), Quantification of lacunar channel depth from (C-F), n = 20 cells. (G-J) Slit diaphragm morphology appears similar in wild type controls (G), *Syx17^{LL} and Plekhm1^{d18L}* single (H, I, respectively) and *Plekhm1^{d18}; Syx17^{LL}* double mutants (J) as shown by anti-Sns and anti-Pyd staining. Cortical sections (G-J) show diaphragm patterns at the cell surface and G^1-J^1 show higher magnifications of the areas in cyan rectangles. Medial sections (g-j for Sns and g^1-j^1 for Pyd) show deeper regions of nephrocytes.

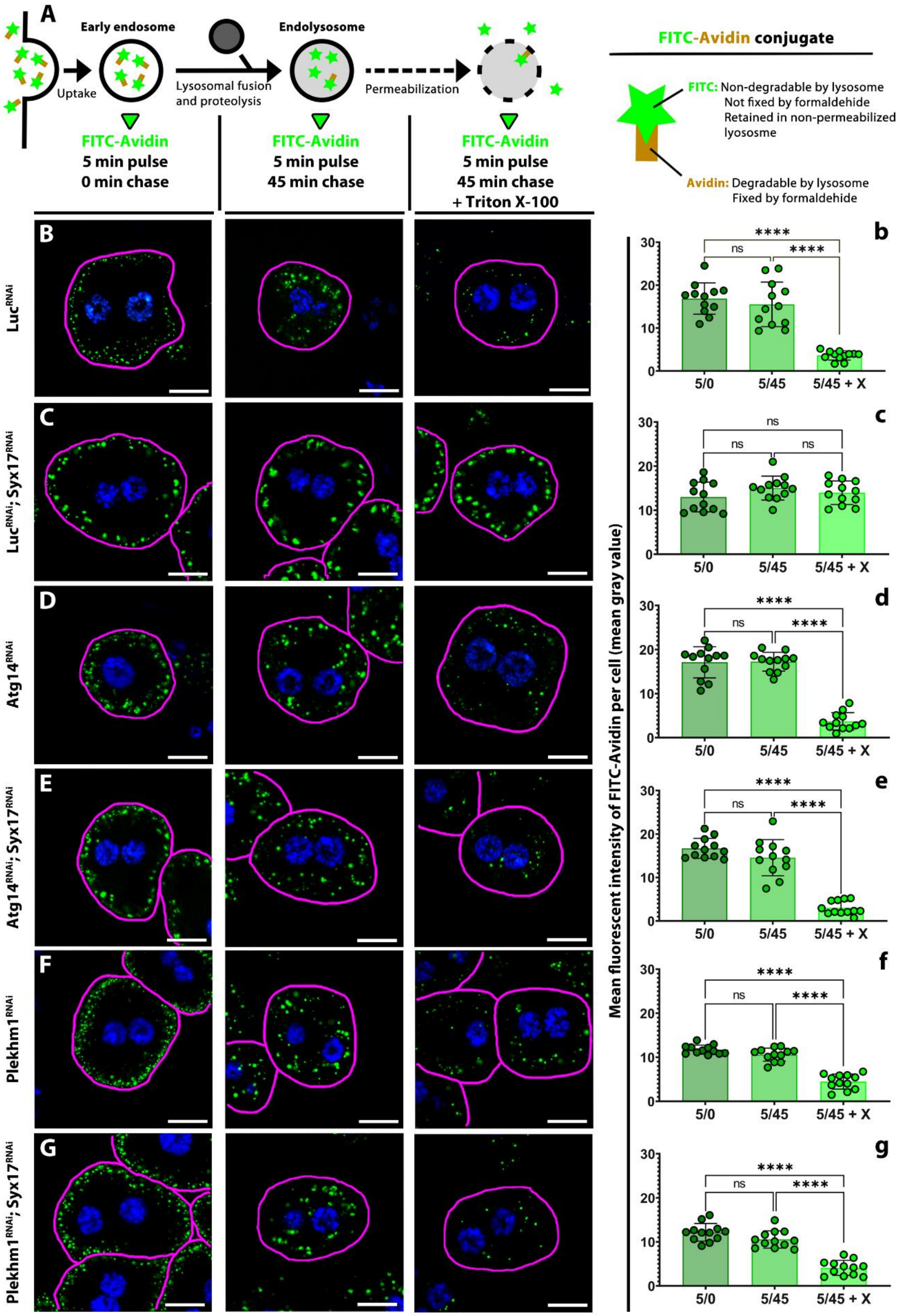
A
Early endosome
Uptake
Lysosomal fusion and proteolysis
Endolysosome
Permeabilization
FITC-Avidin
5 min pulse
0 min chase
FITC-Avidin
5 min pulse
45 min chase
FITC-Avidin
5 min pulse
45 min chase
+ Triton X-100
FITC-Avidin conjugate
FITC: Non-degradable by lysosome
Not fixed by formaldehyde
Retained in non-permeabilized lyososme
Avidin: Degradable by lysosome
Fixed by formaldehyde
B
C
D
E
F
G
Luc^RNAi
Luc^RNAi; Syx17^RNAi
Atg14^RNAi
Atg14^RNAi; Syx17^RNAi
Plekhm1^RNAi
Plekhm1^RNAi; Syx17^RNAi
b
c
d
e
f
g
Mean fluorescent intensity of FITC-Avidin per cell (mean gray value)
5/0
5/45
5/45 + X
ns
****

Fig. S9. Endolysosomes cannot degrade cargo in Syx17 RNAi cells, but this effect can be rescued by Atg14 or Plekhm1 RNAi.

(A): Scheme of the uptake and degradation essay described in Materials and Methods. Briefly: nephrocytes were incubated with the tracer for 5 min and were fixed and imaged immediately (5 min. pulse, 0. min chase) or were chased for 45 min., then fixed. Half of the chased samples were left intact, while the other half was treated with detergent (0.1% Triton-X 100 in PBS) overnight at 4°C followed by washing with PBS (three times for 10 at RT). (B-F): In control cells (A), the tracer was transported into large vesicular structures (presumably lysosomes) during the chase. The signal of the tracer was significantly decreased after treating the cells with TritonX-100, indicating that the uptaken tracer underwent lysosomal degradation and the resulting smaller tracer fragments could be washed out from the permeabilized endolysosomes. In all examined RNAi cases: Luciferase-Syx17 double RNAi (B), Atg14 RNAi (C), Atg14-Syx17 double RNAi (D), Plekhm1 RNAi (E), and Plekhm1-Syx17 double RNAi (F), the tracer could be internalized and transported into larger structures. However, in contrast to others, detergent treatment and subsequent washing could not remove the tracer from Luciferase-Syx17 double RNAi cells, indicating that endolysosomal degradation is reduced upon Syx17 depletion. Blue channel shows the nuclei of the cells (stained by DAPI). The outline of the cells is indicated by magenta lines. Scale bars: 10 µm. (b – g) Quantifications of the mean fluorescent intensity (mean gray value) data in B–G respectively, n = 12 cells. ns: $p>0.05$, ****: $p<0.0001$.

Table S1. List of the genotypes of animals used in this study

Figure		Drosophila melanogaster genotype
1.	A	white[1118]/(y); UAS-DCR2/+; UAS-Luciferase[JF01355]/Prospero-Gal4
	B	white[1118]/(y); UAS-DCR2/+; UAS-Luciferase[JF01355]/UAS-Syntaxin17[JF01937],Prospero-Gal4
	C	white[1118]/(y); UAS-DCR2/+; UAS-Atg14[KK100903]/Prospero-Gal4
	D	white[1118]/(y); UAS-DCR2/+; UAS-Atg14[KK100903]/UAS-Syntaxin17[JF01937],Prospero-Gal4
	F	white[1118]/(y); UAS-GFP-myc-2xFYVE/+; UAS-Luciferase[JF01355]/Prospero-Gal4
	G	white[1118]/(y); UAS-GFP-myc-2xFYVE/+; UAS-Luciferase[JF01355]/UAS-Syntaxin17[JF01937],Prospero-Gal4
	H	white[1118]/(y); UAS-GFP-myc-2xFYVE/+; UAS-Atg14[KK100903]/Prospero-Gal4
	I	white[1118]/(y); UAS-GFP-myc-2xFYVE/+; UAS-Atg14[KK100903]/UAS-Syntaxin17[JF01937],Prospero-Gal4
	K	white[1118]/(y); UAS-DCR2/+; UAS-Luciferase[JF01355]/Prospero-Gal4
	L	white[1118]/(y); UAS-DCR2/+; UAS-Luciferase[JF01355]/UAS-Syntaxin17[JF01937],Prospero-Gal4
	M	white[1118]/(y); UAS-DCR2/+; UAS-Atg14[KK100903]/Prospero-Gal4
	N	white[1118]/(y); UAS-DCR2/+; UAS-Atg14[KK100903]/UAS-Syntaxin17[JF01937],Prospero-Gal4
	Q	white[1118]/(y); UAS-DCR2/+; UAS-Luciferase[JF01355]/Prospero-Gal4
	R	white[1118]/(y); UAS-DCR2/+; UAS-Luciferase[JF01355]/UAS-Syntaxin17[JF01937],Prospero-Gal4
S1.	A	white[1118]/(y); UAS-DCR2/+; UAS-Syx17[GD14850]/Prospero-Gal4
	B	white[1118]/(y); UAS-DCR2/+; UAS-Atg1[GD7149]/Prospero-Gal4
	C	white[1118]/(y); UAS-DCR2/+; UAS-Atg1[GD7149]/UAS-Syntaxin17[JF01937],Prospero-Gal4
	D	white[1118]
	E	white[1118]; +; Syntaxin17[LL06330]
	F	white[1118]/(y); +/UAS-Syx17.Z.EGFP; Syntaxin17[LL06330]/+

	I	white[1118]/(y); UAS-RFP-KDEL/+; UAS-Luciferase[JF01355]/Prospero-Gal4
	J	white[1118]/(y); UAS-RFP-KDEL/+; UAS-Syntaxin17[JF01937]/Prospero-Gal4
	L	white[1118]/(y); Sns-sfGFP/+; UAS-Luciferase[JF01355]/Prospero-Gal4
	M	white[1118]/(y); Sns-sfGFP/+; UAS-Syntaxin17[JF01937]/Prospero-Gal4
	N	white[1118]/(y); UAS-DCR2/+; UAS-Luciferase[JF01355]/Prospero-Gal4
	O	white[1118]/(y); UAS-DCR2/+; UAS-Syntaxin17[JF01937]/Prospero-Gal4
	P	white[1118]/(y); UAS-DCR2/+; UAS-Luciferase[JF01355]/Prospero-Gal4
	Q	white[1118]/(y); UAS-DCR2/+; UAS-Syntaxin17[JF01937]/Prospero-Gal4
	S	white[1118]/(y); UAS-DCR2/+; UAS-Luciferase[JF01355]/Prospero-Gal4
	T	white[1118]/(y); UAS-DCR2/+; UAS-Syntaxin17[JF01937]/Prospero-Gal4
	V	white[1118]/(y); UAS-DCR2/+; UAS-Luciferase[JF01355]/Prospero-Gal4
	W	white[1118]/(y); UAS-DCR2/+; UAS-Syntaxin17[JF01937]/Prospero-Gal4
**S2.**	A	white[1118]/(y); UAS-DCR2/UAS-Syx17.Z.EGFP; UAS-Luciferase[JF01355]/Prospero-Gal4
	B	white[1118]/(y); UAS-DCR2/UAS-Syx17.Z.EGFP; UAS-Vps16A[GD13782]/Prospero-Gal4
	C	white[1118]/(y); UAS-DCR2/+; UAS-Syx17[GD14850]/Prospero-Gal4
	D	white[1118]/(y); UAS-DCR2/+; UAS-Atg1[GD7149]/Prospero-Gal4
	E	white[1118]/(y); UAS-DCR2/+; UAS-Atg1[GD7149]/UAS-Syntaxin17[JF01937],Prospero-Gal4
	F	white[1118]
	G	white[1118]; +; Syntaxin17[LL06330]
	H	white[1118]/(y); +/UAS-Syx17.Z.EGFP; Syntaxin17[LL06330]/+
**S3.**	A	white[1118]/(y); UAS-DCR2/+; UAS-Luciferase[JF01355]/Prospero-Gal4
	B	white[1118]/(y); UAS-DCR2/+; UAS-Syntaxin17[JF01937],Prospero-Gal4/+
	C	white[1118]/(y); UAS-DCR2/+; Prospero-Gal4/UAS-Snap29[JF01883]
	D	white[1118]/(y); UAS-DCR2/UAS-Snap29[HMC03467] (FBst0051893); Prospero-Gal4/+

	E	white[1118]/(y); UAS-DCR2/UAS-Snap29[KK108034]; Prospero-Gal4/+
	F	white[1118]/(y); UAS-DCR2/UAS-Vamp7[1599R-1]; Prospero-Gal4/+
	G	white[1118]/(y); UAS-DCR2/+; Prospero-Gal4/UAS-Vamp7[GL01524]
	H	white[1118]/(y); UAS-DCR2/+; Prospero-Gal4/UAS-Rab2[GD11158]
	I	white[1118]/(y); UAS-DCR2/+; Prospero-Gal4/UAS-Rab7[JF02377]
	J	white[1118]/(y); UAS-DCR2/+; Prospero-Gal4/UAS-Arl8[11926R-1]
	K	white[1118]/(y); UAS-DCR2/+; Prospero-Gal4/UAS-Vps41[3093R-2]
	L	white[1118]/(y); UAS-DCR2/UAS-Vps11[KK102566]; Prospero-Gal4/+
**2.**	A	white[1118]/(y); UAS-DCR2/+; UAS-Syntaxin17[JF01937],Prospero-Gal4/+
	B	
	C	white[1118]/(y); UAS-DCR2/UAS-Arl8-GFP; UAS-Syntaxin17[JF01937],Prospero-Gal4/+
	D	gen-Vps8-9xHA/(y); UAS-DCR2/+; UAS-Syntaxin17[JF01937],Prospero-Gal4/+
	E	white[1118]/(y); UAS-Arl8-GFP/+; UAS-Syntaxin17[JF01937],Prospero-Gal4/UAS-Vps41-9xHA
	F	white[1118]/(y); UAS-DCR2/UAS-Vamp7.GFP; UAS-Syntaxin17[JF01937],Prospero-Gal4/+
	G	white[1118]/(y); UAS-DCR2/3xmCherry-Atg8a; UAS-Syntaxin17[JF01937],Prospero-Gal4/+
**S4.**	A	white[1118]/(y); UAS-DCR2/+; UAS-Syntaxin17[JF01937],Prospero-Gal4/+
	B	white[1118]/(y); UAS-DCR2/UAS-2xEGFP; UAS-Syntaxin17[JF01937],Prospero-Gal4/+
	C	white[1118]/(y); UAS-DCR2/+; UAS-Syntaxin17[JF01937],Prospero-Gal4/+
	D	
	E	white[1118]/(y); UAS-DCR2/+; UAS-Syntaxin17[JF01937],Prospero-Gal4/UAS-Rbsn-5.ORF.3xHA(FBst0501500)
	F	white[1118]/(y); UAS-DCR2/+; UAS-Syntaxin17[JF01937],Prospero-Gal4/UAS-Past1.B.GFP (FBst0081056)
	G	white[1118]/(y); UAS-DCR2/UAS-GFP.Sec15 (FBst0039685); UAS-Syntaxin17[JF01937],Prospero-Gal4/+
**4.**	A	white[1118]/(y); UAS-DCR2/+; UAS-Luciferase[JF01355]/UAS-Syntaxin17[JF01937],Prospero-Gal4

	B	white[1118]/(y); UAS-DCR2/UAS-Vamp7[1599R-1]; UAS-Syntaxin17[JF01937],Prospero-Gal4/+
	C	white[1118]/(y); UAS-DCR2/UAS-Vamp7[1599R-1]; UAS-Syntaxin17[JF01937],Prospero-Gal4/UAS-Snap29[JF01883]
	D	white[1118]/(y); UAS-DCR2/+; UAS-Syntaxin17[JF01937],Prospero-Gal4/UAS-Rab7[JF02377]
	E	white[1118]/(y); UAS-DCR2/+; UAS-Syntaxin17[JF01937],Prospero-Gal4/UAS-Rab2[GD11158]
	F	white[1118]/(y); UAS-DCR2/UAS-Vps11[KK102566]; UAS-Syntaxin17[JF01937],Prospero-Gal4/+
**S5.**	A	white[1118]/(y); UAS-DCR2/+; UAS-Syntaxin17[JF01937],Prospero-Gal4/UAS-Snap29[JF01883]
	B	white[1118]/(y); UAS-DCR2/+; UAS-Syntaxin17[JF01937],Prospero-Gal4/UAS-Rab7[GD11800]
	C	white[1118]/(y); UAS-DCR2/+; UAS-Syntaxin17[JF01937],Prospero-Gal4/UAS-Mon1[11926R-1]
	D	white[1118]/(y); UAS-DCR2/+; UAS-Syntaxin17[JF01937],Prospero-Gal4/UAS-Ccz1[GD8501]
	E	white[1118]/(y); UAS-DCR2/+; UAS-Syntaxin17[JF01937],Prospero-Gal4/UAS-Arl8[11926R-1]
	F	white[1118]/(y); UAS-DCR2/+; UAS-Syntaxin17[JF01937],Prospero-Gal4/UAS-Vps41[3093R-2]
	G	white[1118]/(y); UAS-DCR2/+; UAS-Syntaxin17[JF01937],Prospero-Gal4/UAS-Vps39[GD12152]
	H	white[1118]/(y); UAS-DCR2/+; UAS-Syntaxin17[JF01937],Prospero-Gal4/UAS-Vps33A[GD1397]
	I	white[1118]/(y); UAS-DCR2/Vps18[KK102176]; UAS-Syntaxin17[JF01937],Prospero-Gal4
	J	white[1118]/(y); UAS-DCR2/UAS-Vps16A[GD13782]; UAS-Syntaxin17[JF01937],Prospero-Gal4/+
	K	white[1118]/(y); UAS-DCR2/UAS-Vps8[KK100319]; UAS-Syntaxin17[JF01937],Prospero-Gal4/+
	L	white[1118]/(y); UAS-DCR2/UAS-Vps45[HMS01696]; UAS-Syntaxin17[JF01937],Prospero-Gal4/+
	M	white[1118]/(y); UAS-DCR2/UAS-Vps33B[HMS02720]; UAS-Syntaxin17[JF01937],Prospero-Gal4/+
	N	white[1118]/(y); UAS-DCR2/UAS-Rbsn5[HMC04769]; UAS-Syntaxin17[JF01937],Prospero-Gal4/+
	O	white[1118]/(y); UAS-DCR2/+; UAS-Syntaxin17[JF01937],Prospero-Gal4/UAS-Sec5[JF02676]
	P	white[1118]/(y); UAS-DCR2/+; UAS-Syntaxin17[JF01937],Prospero-Gal4/UAS-Past1[HMS00557]

5.	A	white[1118]/(y); UAS-DCR2/+; UAS-Luciferase[JF01355]/Prospero-Gal4,UAS-Rab7[JF02377]
	B	white[1118]/(y); UAS-Vamp7[1599R-1]/+; UAS-Luciferase[JF01355]/Prospero-Gal4
	C	white[1118]/(y); UAS-DCR2/+; UAS-Sec5[JF02676]/Prospero-Gal4
	D	white[1118]/(y); UAS-DCR2/+; UAS-Sec5[JF02676]/Prospero-Gal4,UAS-Rab7[JF02377]
	E	white[1118]/(y); UAS-Vamp7[1599R-1]/+; UAS-Sec5[JF02676]/Prospero-Gal4
	H	white[1118]
	I	white[1118]; +; Syntaxin17[LL06330]
	J	white[1118]; +; Vps16A[d32]
	K	white[1118]; +; Vps16A[d32], Syntaxin17[LL06330]
S6.	A	white[1118]/(y); UAS-DCR2/+; UAS-Sec15[JF02649]/Prospero-Gal4
	B	white[1118]/(y); UAS-DCR2/+; UAS-Sec15[JF02649]/Prospero-Gal4,UAS-Rab7[JF02377]
	C	white[1118]/(y); UAS-Vamp7[1599R-1]/+; UAS-Sec15[JF02649]/Prospero-Gal4
	D	white[1118]/(y); UAS-Vamp7[1599R-1]/+; UAS-Sec5[JF02676]/Prospero-Gal4
	E	white[1118]; +; Syntaxin17[LL06330]
	F	
6.	A	white[1118]/(y); UAS-DCR2/+; UAS-Luciferase[JF01355]/Prospero-Gal4
	B	white[1118]/(y); UAS-DCR2/Plekhm1[GD11978]; Prospero-Gal4/+
	C	white[1118]/(y); UAS-DCR2/+; UAS-Luciferase[JF01355]/UAS-Syntaxin17[JF01937],Prospero-Gal4
	D	white[1118]/(y); UAS-DCR2/Plekhm1[GD11978]; UAS-Syntaxin17[JF01937],Prospero-Gal4/+
	E	white[1118]/(y); UAS-DCR2/+; UAS-Luciferase[JF01355]/Prospero-Gal4
	F	white[1118]/(y); UAS-DCR2/Plekhm1[GD11978]; Prospero-Gal4/+
	G	white[1118]/(y); UAS-DCR2/+; UAS-Luciferase[JF01355]/UAS-Syntaxin17[JF01937],Prospero-Gal4
	H	white[1118]/(y); UAS-DCR2/Plekhm1[GD11978]; UAS-Syntaxin17[JF01937],Prospero-Gal4/+

**S7.**	A	white[1118]/(y); UAS-DCR2/UAS-Def8[11534R-3]; Prospero-Gal4/+
	C	white[1118]/(y); UAS-DCR2/+; UAS-Luciferase[JF01355]/Prospero-Gal4, UAS-Vps41-9xHA
	D	white[1118]/(y); UAS-DCR2/Plekhm1[GD11978]; Prospero-Gal4, UAS-Vps41-9xHA/+
	E	white[1118]
	F	white[1118]; +; Syntaxin17[LL06330]
	G	white[1118]; plekhm1[d18]; +
	H	white[1118]; plekhm1[d18]; Syntaxin17[LL06330]
	I	white[1118]
	J	white[1118]; +; Syntaxin17[LL06330]
	K	white[1118]; plekhm1[d18]; +
	L	white[1118]; plekhm1[d18]; Syntaxin17[LL06330]
**7.**	A	white[1118]/(y); UAS-DCR2/+; UAS-Syntaxin17[JF01937],Prospero-Gal4/+
	B	white[1118]/(y); UAS-DCR2/Plekhm1[GD11978]; UAS-Syntaxin17[JF01937],Prospero-Gal4/+
	C	white[1118]
	D	white[1118]; plekhm1[d18]; +
	E	white[1118]; +; Syntaxin17[LL06330]
	F	white[1118]; plekhm1[d18]; Syntaxin17[LL06330]
**S8.**	A	white[1118]; plekhm1[d18]; Syntaxin17[LL06330]
	C	white[1118]
	D	white[1118]; +; Syntaxin17[LL06330]
	E	white[1118]; plekhm1[d18]; +
	F	white[1118]; plekhm1[d18]; Syntaxin17[LL06330]
	G	white[1118]
	H	white[1118]; +; Syntaxin17[LL06330]

	I	white[1118]; plekhm1[d18]; +
	J	white[1118]; plekhm1[d18]; Syntaxin17[LL06330]
**8.**	A	white[1118]/(y); UAS-DCR2/+; UAS-Luciferase[JF01355]/Prospero-Gal4, UAS-Vps41-9xHA
	B	white[1118]/(y); UAS-DCR2/+; UAS-Luciferase[JF01355],UAS-Syntaxin17[JF01937]/Prospero-Gal4, UAS-Vps41-9xHA
	C	white[1118]/(y); UAS-DCR2/+; UAS-Atg14[KK100903]/Prospero-Gal4, UAS-Vps41-9xHA
	D	white[1118]/(y); UAS-DCR2/+; UAS-Atg14[KK100903],UAS-Syntaxin17[JF01937]/Prospero-Gal4, UAS-Vps41-9xHA
	E	white[1118]/(y); UAS-DCR2/Plekhm1[GD11978]; Prospero-Gal4, UAS-Vps41-9xHA/+
	F	white[1118]/(y); UAS-DCR2/Plekhm1[GD11978]; UAS-Syntaxin17[JF01937]/Prospero-Gal4, UAS-Vps41-9xHA/+
**S9.**	B	white[1118]/(y); UAS-DCR2/+; UAS-Luciferase[JF01355]/Prospero-Gal4
	C	white[1118]/(y); UAS-DCR2/+; UAS-Luciferase[JF01355]/UAS-Syntaxin17[JF01937],Prospero-Gal4
	D	white[1118]/(y); UAS-DCR2/+; UAS-Atg14[KK100903]/Prospero-Gal4
	E	white[1118]/(y); UAS-DCR2/+; UAS-Atg14[KK100903]/UAS-Syntaxin17[JF01937],Prospero-Gal4
	F	white[1118]/(y); UAS-DCR2/Plekhm1[GD11978]; Prospero-Gal4/+
	G	white[1118]/(y); UAS-DCR2/Plekhm1[GD11978]; UAS-Syntaxin17[JF01937],Prospero-Gal4/+

Table S2. Details about the data sets, statistical analyses and P-values.

Figure 1

Panel E

Measured: Rab7 positive endosome size (square micron)
Statistical test: Kruskal-Wallis test with Dunn's multiple comparisons test

Genotype	N	Mean	Standard deviation	P-value	
				Compared to Luc Ri	Compared to Syx17 Ri; Luc Ri
Luc Ri	442	1.52	0.78	-	<0,0001
Syx17 Ri; Luc Ri	172	4.72	2.70	<0,0001	-
Atg14 Ri	325	1.35	0.66	0.1073	<0,0001
Syx17 Ri; Atg14 Ri	344	1.46	0.76	>0,9999	<0,0001

Panel J

Measured: FYVE-GFP positive endosome size (square micron)
Statistical test: Kruskal-Wallis test with Dunn's multiple comparisons test

Genotype	N	Mean	Standard deviation	P-value	
				Compared to Luc Ri	Compared to Syx17 Ri; Luc Ri
Luc Ri	480	1.27	0.71	-	<0,0001
Syx17 Ri; Luc Ri	250	5.77	4.90	<0,0001	-
Atg14 Ri	339	1.38	0.71	0.0582	<0,0001
Syx17 Ri; Atg14 Ri	370	1.38	0.67	0.0141	<0,0001

Panel O

Measured: Atg8a positive cell area fraction (%)
Statistical test: Kruskal-Wallis test with Dunn's multiple comparisons test

Genotype	N	Mean	Standard deviation	P-value	
				Compared to Luc Ri	Compared to Syx17 Ri; Luc Ri
Luc Ri	20	0.27	0.2	-	<0,0001
Syx17 Ri; Luc Ri	20	5.56	1.74	<0,0001	-
Atg14 Ri	20	0.13	0.14	0.2203	<0,0001
Syx17 Ri; Atg14 Ri	20	0.15	0.12	>0,9999	<0,0001

Panel P

Measured: p62 positive cell area fraction (%)

Statistical test:	Ordinary one-way ANOVA with Tukey's multiple comparisons test				
**Genotype**	**N**	**Mean**	**Standard deviation**	**P-value**	
				**Compared to Luc Ri**	**Compared to Syx17 Ri; Luc Ri**
Luc Ri	20	0.26	0.154	-	<0,0001
Syx17 Ri; Luc Ri	20	4.16	1.78	<0,0001	-
Atg14 Ri	20	3.35	1.45	<0,0001	0.2678
Syx17 Ri; Atg14 Ri	20	3.4	1.59	<0,0001	0.3302

Figure S1

Panel G

Measured: Rab7 positive endosome size (square micron)
Statistical test: Kruskal-Wallis test with Dunn's multiple comparisons test

**Genotype**	**N**	**Mean**	**Standard deviation**	**P-value**	
				**Compared to Luc Ri**	**Compared to Luc Ri; Syx17 Ri**
Luc Ri	442	1.52	0.78	-	<0,0001
Syx17 Ri 2.	172	4.38	3.17	<0,0001	0.0744
Syx17 Ri; Luc Ri	172	4.72	2.70	<0,0001	-
Atg1 Ri	440	1.38	0.69	0.3779	<0,0001
Syx17 Ri; Atg1 Ri	474	1.46	0.88	0.3459	<0,0001

Panel H

Measured: Rab7 positive endosome size (square micron)
Statistical test: Kruskal-Wallis test with Dunn's multiple comparisons test

**Genotype**	**N**	**Mean**	**Standard deviation**	**P-value**	
				**Compared to w1118**	**Compared to Syx17LL**
w1118	365	1.45	0.81	-	<0,0001
Syx17 LL	248	5.20	3.84	<0,0001	-
Syx17 LL; Syx17-GFP	333	1.71	0.90	0.0023	<0,0001

Panel K

Measured: KDEL-RFP positive area of the cell (%)
Statistical test: Unpaired T-test

**Genotype**	**N**	**Mean**	**Standard deviation**	**P-value**	
				**Compared to Luc Ri**	**Compared to Syx17 Ri**
Luc Ri	20	19.38	4.59	-	0.0145
Syx17 Ri	20	15.64	4.65	0.0145	-

Panel R

**Measured:**	Average lacunae depth per cell (micron)				
**Statistical test:**	Unpaired T-test				

Genotype	N	Mean	Standard deviation	P-value Compared to Luc Ri	P-value Compared to Syx17 Ri
Luc Ri	20	1.339	0.136	-	0.5428
Syx17 Ri	20	1.368	0.167	0.5428	-

Panel U

**Measured:**	Average a-cubulin signal depth per cell (micron)				
**Statistical test:**	Unpaired T-test				

Genotype	N	Mean	Standard deviation	P-value Compared to Luc Ri	P-value Compared to Syx17 Ri
Luc Ri	20	1.417	0.170	-	0.9707
Syx17 Ri	20	1.415	0.172	0.9707	-

Figure S2

Panel I

**Measured:**	Atg8a positive cell area fraction (%)				
**Statistical test:**	Ordinary one-way ANOVA with Tukey's multiple comparisons test				

Genotype	N	Mean	Standard deviation	P-value Compared to Luc Ri	P-value Compared to Luc Ri; Syx17 Ri
Luc Ri	20	0.24	0.15	-	<0,0001
Syx17 Ri 2.	20	4.45	1.21	<0,0001	0.8759
Syx17 Ri; Luc Ri	20	4.16	1.78	<0,0001	-
Atg1 Ri	20	0.15	0.13	0.9988	<0,0001
Syx17 Ri; Atg1 Ri	20	0.74	0.35	0.4814	<0,0001

Panel J

**Measured:**	p62 positive cell area fraction (%)				
**Statistical test:**	Ordinary one-way ANOVA with Tukey's multiple comparisons test				

Genotype	N	Mean	Standard deviation	P-value Compared to Luc Ri	P-value Compared to Luc Ri; Syx17 Ri
Luc Ri	20	0.25	0.15	-	<0,0001
Syx17 Ri 2.	20	3.21	1.12	<0,0001	0.0514

Syx17 Ri; Luc Ri	20	4.16	1.78	<0,0001	-
Atg1 Ri	20	3.18	1.05	<0,0001	0.0410
Syx17 Ri; Atg1 Ri	20	1.90	0.59	<0,0001	<0,0001

Panel K

Measured: Atg8a positive cell area fraction (%)
Statistical test: Kruskal-Wallis test with Dunn's multiple comparisons test

Genotype	N	Mean	Standard deviation	P-value Compared to w1118	P-value Compared to Syx17LL
w1118	20	0.24	0.16	-	<0,0001
Syx17 LL	20	4.88	1.92	<0,0001	-
Syx17 LL; Syx17-GFP	20	0.45	0.28	0.2662	<0,0001

Panel L

Measured: p62 positive cell area fraction (%)
Statistical test: Ordinary one-way ANOVA with Tukey's multiple comparisons test

Genotype	N	Mean	Standard deviation	P-value Compared to w1118	P-value Compared to Syx17LL
w1118	20	0.57	0.48	-	<0,0001
Syx17 LL	20	3.87	1.37	<0,0001	-
Syx17 LL; Syx17-GFP	20	0.86	0.46	0.5461	<0,0001

Figure S3

Panel M

Measured: Atg8a positive cell area fraction (%)
Statistical test: Kruskal-Wallis test with Dunn's multiple comparisons test

Genotype	N	Mean	Standard deviation	P-value Compared to Luc Ri	P-value Compared to Syx17 Ri
Luc Ri	20	0.12	0.07	-	<0,0001
Syx17 Ri	20	4.93	1.61	<0,0001	-
Snap29 Ri 1.	20	1.30	0.83	<0,0001	0.0491
Snap29 Ri 2.	20	0.40	0.31	0.1308	<0,0001
Snap29 Ri 3.	20	0.67	0.37	<0,0001	<0,0001
Vamp7 Ri 1.	20	0.28	0.22	>0,9999	<0,0001
Vamp7 Ri 2.	20	0.55	0.32	0.0010	<0,0001
Rab2 Ri	20	0.80	0.33	<0,0001	0.035
Rab7 Ri	20	0.67	0.34	<0,0001	0.0001
Arl8 Ri	20	0.33	0.19	0.2936	<0,0001
Vps41 Ri	20	0.51	0.30	0.0024	<0,0001

Vps11 Ri	20	0.99	0.53	<0,0001	0.0163

Panel N

Measured: p62 positive cell area fraction (%)

Statistical test: Kruskal-Wallis test with Dunn's multiple comparisons test

Genotype	N	Mean	Standard deviation	P-value Compared to Luc Ri	P-value Compared to Syx17 Ri
Luc Ri	20	0.30	0.27	-	<0,0001
Syx17 Ri	20	3.43	1.22	<0,0001	-
Snap29 Ri 1.	20	1.59	0.82	<0,0001	0.5161
Snap29 Ri 2.	20	0.40	0.21	>0,9999	<0,0001
Snap29 Ri 3.	20	1.28	0.71	<0,0001	0.0292
Vamp7 Ri 1.	20	0.45	0.28	>0,9999	<0,0001
Vamp7 Ri 2.	20	0.55	0.32	0.3413	<0,0001
Rab2 Ri	20	0.70	0.42	0.0094	<0,0001
Rab7 Ri	20	0.49	0.23	0.6942	<0,0001
Arl8 Ri	20	0.51	0.24	0.4567	<0,0001
Vps41 Ri	20	0.55	0.30	0.2802	<0,0001
Vps11 Ri	20	1.18	0.54	<0,0001	0.039

Figure 2

Panel F

Measured: Cluster-to-cytoplasm ratio

Statistical test: Ordinary one-way ANOVA with Tukey's multiple comparisons test with the transformed data

Genotype	N	Mean	Standard deviation	P-value Compared to 2xEGFP
2xEGFP	30	1.01	0.13	-
Rbsn5-HA	30	0.45	0.15	<0,0001
Vps8-HA	30	0.50	0.24	<0,0001
a-Vps16a	30	2.10	0.58	<0,0001
Vps41-HA	30	7.79	2.49	<0,0001
Past1-GFP	30	0.65	0.21	<0,0001
Sec15-GFP	30	0.64	0.25	<0,0001
a-Cathepsin	30	2.90	1.00	<0,0001
a-Lamp1	30	6.13	2.55	<0,0001
Arl8-GFP	30	5.28	2.13	<0,0001
Vamp7-GFP	30	5.26	2.18	<0,0001
a-SNAP29	30	2.65	0.94	<0,0001

Figure 3

Panel E

**Measured:**	Average distance (micron) between an LCB+ vesicle and its five nearest LC3B+ vesicles
**Statistical test:**	Kruskal-Wallis test with Dunn's multiple comparisons test

Genotype	N	Mean	Standard deviation	P-value Compared to Control	P-value Compared to siSTX17
Control	100	2.234	0.770	-	<0,0001
siSTX17	100	0.801	0.249	<0,0001	-
siSTX17, STX17(WT)	100	2.088	0.695	>0,9999	<0,0001
siSTX17, STX17(R/K>A)	100	1.062	0.386	<0,0001	0.0052

Panel G

**Measured:**	Protein level of STX17 (normalized for TUB level)
**Statistical test:**	Mann-Whitney test

Genotype	N	Mean	Standard deviation	P-value Compared to Control	P-value Compared to siSTX17
Control	6	1.002	0.104	-	0.0022
siSTX17	6	0.330	0.137	0.0022	-

Figure 4

Panel G

**Measured:**	Atg8a positive cell area fraction (%)
**Statistical test:**	Kruskal-Wallis test with Dunn's multiple comparisons test

Genotype	N	Mean	Standard deviation	P-value Compared to Luc Ri; Syx17 Ri
Luc Ri; Syx17 Ri	20	4.68	1.36	-
Snap29 Ri; Syx17 Ri	20	4.49	1.44	>0,9999
Vamp7 Ri; Syx17 Ri	20	4.52	1.54	>0,9999
Snap29 Ri; Vamp7 Ri; Syx17 Ri	20	3.96	1.46	>0,9999
Rab7 Ri 1; Syx17 Ri	20	0.61	0.29	<0,0001
Rab7 Ri 2; Syx17 Ri	20	1.00	0.49	<0,0001
Mon1 Ri; Syx17 Ri	20	0.71	0.37	<0,0001
Cccz1 Ri; Syx17 Ri	20	0.76	0.29	<0,0001
Arl8 Ri; Syx17 Ri	20	0.88	0.44	<0,0001
Rab2 Ri; Syx17 Ri	20	1.13	0.44	<0,0001
Vps41 Ri; Syx17 Ri	20	0.70	0.27	<0,0001
Vps11 Ri; Syx17 Ri	20	1.38	0.74	<0,0001

Vps39 Ri; Syx17 Ri	20	1.14	0.59	<0,0001
Vps33a Ri; Syx17 Ri	20	0.82	0.39	<0,0001
Vps18 Ri; Syx17 Ri	20	0.83	0.44	<0,0001
Vps16 Ri; Syx17 Ri	20	0.99	0.47	<0,0001
Vps8 Ri; Syx17 Ri	20	4.40	1.66	>0,9999
Vps45 Ri; Syx17 Ri	20	4.30	1.22	>0,9999
Vps33b Ri; Syx17 Ri	20	3.59	1.03	>0,9999
Rbsn5 Ri; Syx17 Ri	20	3.12	0.63	>0,9999
Sec5 Ri; Syx17 Ri	20	4.27	1.45	>0,9999
Past1 Ri; Syx17 Ri	20	4.34	0.88	>0,9999

Figure 5

Panel F

Measured: Atg8a positive cell area fraction (%)
Statistical test: Kruskal-Wallis test with Dunn's multiple comparisons test

Genotype	N	Mean	Standard deviation	P-value Compared to Rab7 Ri; Luc Ri	P-value Compared to Vamp7 Ri; Luc Ri
Rab7 Ri; Luc Ri	20	0.740	0.336	-	>0,9999
Vamp7 Ri; Luc Ri	20	0.395	0.329	>0,9999	-
Sec5 Ri	20	0.472	0.252	>0,9999	>0,9999
Sec15 Ri	20	0.401	0.390	>0,9999	>0,9999
Rab7 Ri; Sec5 Ri	20	4.261	1.373	<0,0001	<0,0001
Rab7 Ri; Sec15 Ri	20	3.525	1.327	0.0023	<0,0001
Vamp7 Ri; Sec5 Ri	20	4.084	1.427	0.0001	<0,0001
Vamp7 Ri; Sec15 Ri	20	3.894	1.552	0.0006	<0,0001

Panel G

Measured: p62 positive cell area fraction (%)
Statistical test: Kruskal-Wallis test with Dunn's multiple comparisons test

Genotype	N	Mean	Standard deviation	P-value Compared to Rab7 Ri; Luc Ri	P-value Compared to Vamp7 Ri; Luc Ri
Rab7 Ri; Luc Ri	20	0.740	0.336	-	0.9104
Vamp7 Ri; Luc Ri	20	0.395	0.329	0.9104	-
Sec5 Ri	20	0.472	0.252	>0,9999	>0,9999
Sec15 Ri	20	0.401	0.390	>0,9999	>0,9999
Rab7 Ri; Sec5 Ri	20	4.261	1.373	0.0001	<0,0001
Rab7 Ri; Sec15 Ri	20	3.525	1.327	0.0008	<0,0001
Vamp7 Ri; Sec5 Ri	20	4.084	1.427	0.0015	<0,0001

Vamp7 Ri; Sec15 Ri	20	3.894	1.552	0.0001	<0,0001

Figure 6

Panel I

Measured: Rab7 positive endosome size (square micron)
Statistical test: Kruskal-Wallis test with Dunn's multiple comparisons test

Genotype	N	Mean	Standard deviation	P-value Compared to Luc Ri	P-value Compared to Syx17 Ri; Luc Ri
Luc Ri	343	1.57	0.83	-	<0,0001
Plekhm1 Ri	371	1.62	0.97	>0,9999	<0,0001
Syx17 Ri; Luc Ri	276	4.9	3.14	<0,0001	-
Syx17 Ri; Plekhm1 Ri	393	1.64	1.04	>0,9999	<0,0001

Panel J

Measured: Atg8a positive cell area fraction (%)
Statistical test: Ordinary one-way ANOVA with Tukey's multiple comparisons test

Genotype	N	Mean	Standard deviation	P-value Compared to Luc Ri	P-value Compared to Syx17 Ri; Luc Ri
Luc Ri	20	0.18	0.10	-	<0,0001
Plekhm1 Ri	20	0.55	0.27	0.3645	<0,0001
Syx17 Ri; Luc Ri	20	5.14	1.28	<0,0001	-
Syx17 Ri; Plekhm1 Ri	20	1.59	0.59	<0,0001	<0,0001

Panel K

Measured: p62 positive cell area fraction (%)
Statistical test: Ordinary one-way ANOVA with Tukey's multiple comparisons test

Genotype	N	Mean	Standard deviation	P-value Compared to Luc Ri	P-value Compared to Syx17 Ri; Luc Ri
Luc Ri	20	0.35	0.16	-	<0,0001
Plekhm1 Ri	20	1.62	0.71	<0,0001	<0,0001
Syx17 Ri; Luc Ri	20	5.08	1.46	<0,0001	-
Syx17 Ri; Plekhm1 Ri	20	1.36	0.59	0.0023	<0,0001

Figure S7

Panel N

Measured: Rab7 positive endosome size (square micron)
Statistical test: Kruskal-Wallis test with Dunn's multiple comparisons test

Genotype	N	Mean	Standard deviation	P-value Compared to w1118	P-value Compared to Syx17LL
w1118	402	1.47	0.84	-	<0,0001
Syx17 LL	277	4.13	3.56	<0,0001	-
Plekhm1 d18	384	1.46	0.80	>0,9999	<0,0001
Syx17 LL; Plekhm1 d18	408	1.79	1.32	0.4119	<0,0001

Panel O

Measured: Atg8a positive cell area fraction (%)
Statistical test: Kruskal-Wallis test with Dunn's multiple comparisons test

Genotype	N	Mean	Standard deviation	P-value Compared to w1118	P-value Compared to Syx17LL
w1118	20	0.13	0.08	-	<0,0001
Syx17 LL	20	4.71	1.09	<0,0001	-
Plekhm1 d18	20	0.25	0.17	>0,9999	<0,0001
Syx17 LL; Plekhm1 d18	20	1.04	0.49	<0,0001	0.0282

Panel P

Measured: p62 positive cell area fraction (%)
Statistical test: Kruskal-Wallis test with Dunn's multiple comparisons test

Genotype	N	Mean	Standard deviation	P-value Compared to w1118	P-value Compared to Syx17LL
w1118	20	0.26	0.10	-	<0,0001
Syx17 LL	20	4.25	1.20	<0,0001	-
Plekhm1 d18	20	0.11	0.12	0.1621	<0,0001
Syx17 LL; Plekhm1 d18	20	0.74	0.37	0.0917	0.0120

Figure 7

Panel G

Measured: Lacunae localized Atg8a positive cell area fraction (%)
Statistical test: Kruskal-Wallis test with Dunn's multiple comparisons test

Genotype	N	Mean	Standard deviation	P-value Compared to w1118
w1118	20	0.068	0.072	-
Syx17 LL	20	0.075	0.072	>0,9999
Plekhm1 d18	20	0.093	0.066	>0,9999
Syx17 LL; Plekhm1 d18	20	0.470	0.240	<0,0001

Figure S8

Panel B

Measured: Average lacunae depth per cell (micron)

Statistical test: Ordinary one-way ANOVA with Tukey's multiple comparisons test

Genotype	N	Mean	Standard deviation	P-value Compared to w1118
w1118	20	1.338	0.209	-
Syx17 LL	20	1.365	0.186	0.9716
Plekhm1 d18	20	1.362	0.158	0.9787
Syx17 LL; Plekhm1 d18	20	1.381	0.207	0.8924

Figure 8

Panel G

Measured: Percentage of peripheral LAMP1 signal (%)

Statistical test: Ordinary one-way ANOVA with Tukey's multiple comparisons test

Genotype	N	Mean	Standard deviation	P-value	
				Compared to Luc Ri	Compared to Luc Ri; Syx17 Ri
Lucc RNAi	10	14.83	5.19	-	<0,0001
Luc RNAi; Syx17 RNAi	10	37.67	5.47	<0,0001	-
Atg14 RNAi	10	14.11	4.44	0.9997	<0,0001
Plekhm1 RNAi	10	18.59	4.92	0.6585	<0,0001
Syx17 RNAi; Atg14 RNAi	10	16.00	6.66	0.997	<0,0001
Syx17 RNAi; Plekhm1 RNAi	10	16.11	6.40	0.9954	<0,0001

Panel H

Measured: Percentage of peripheral Vps41-HA signal (%)

Statistical test: Ordinary one-way ANOVA with Tukey's multiple comparisons test

Genotype	N	Mean	Standard deviation	P-value	
				Compared to Luc Ri	Compared to Luc Ri; Syx17 Ri
Lucc RNAi	10	21.44	4.19	-	<0,0001
Luc RNAi; Syx17 RNAi	10	49.35	7.66	<0,0001	-
Atg14 RNAi	10	19.66	5.58	0.9905	<0,0001
Plekhm1 RNAi	10	21.28	6.50	>0,9999	<0,0001
Syx17 RNAi; Atg14 RNAi	10	21.66	9.13	>0,9999	<0,0001

Syx17 RNAi; Plekhm1 RNAi	10	21.38	5.55	>0,9999	<0,0001

Panel I

Measured: LAMP1 ~ Vps41-HA: Pearson's coefficient

Statistical test: Ordinary one-way ANOVA with Tukey's multiple comparisons test

Genotype	N	Mean	Standard deviation	P-value Compared to Luc Ri	P-value Compared to Luc Ri; Syx17 Ri
Lucc RNAi	10	0.275	0.0369	-	<0,0001
Luc RNAi; Syx17 RNAi	10	0.482	0.0643	<0,0001	-
Atg14 RNAi	10	0.340	0.0523	0.0986	<0,0001
Plekhm1 RNAi	10	0.299	0.0637	0.9207	<0,0001
Syx17 RNAi; Atg14 RNAi	10	0.357	0.0529	0.0168	<0,0001
Syx17 RNAi; Plekhm1 RNAi	10	0.371	0.0522	0.0031	0.0004

Figure S9

Panel b - Luc RNAi

Measured: Mean fluorescent intensity of FITC-Avidin per cell (Mean grey value)

Statistical test: Ordinary one-way ANOVA with Tukey's multiple comparisons test

Experiment	N	Mean	Standard deviation	P-value Compared to 5,0	P-value Compared to 5,45
5 pulse 0 chase	12	16.90	3.63	-	0.6468
5 pulse 45 chase	12	15.50	5.19	0.6468	-
5 pulse 45 chase + TritonX	12	3.63	1.06	<0,0001	<0,0001

Panel c - Luc RNAi; Syx17 RNAi

Measured: Mean fluorescent intensity of FITC-Avidin per cell (Mean grey value)

Statistical test: Ordinary one-way ANOVA with Tukey's multiple comparisons test

Experiment	N	Mean	Standard deviation	P-value Compared to 5,0	P-value Compared to 5,45
5 pulse 0 chase	12	13.00	3.37	-	0.2247
5 pulse 45 chase	12	15.00	2.71	0.2247	-
5 pulse 45 chase + TritonX	12	13.90	2.68	0.7106	0.6463

Panel d - Atg14 RNAi

**Measured:**	Mean fluorescent intensity of FITC-Avidin per cell (Mean grey value)				
**Statistical test:**	Ordinary one-way ANOVA with Tukey's multiple comparisons test				
**Experiment**	**N**	**Mean**	**Standard deviation**	**P-value** Compared to 5,0	**P-value** Compared to 5,45
5 pulse 0 chase	12	17.10	3.53	-	0.9911
5 pulse 45 chase	12	17.30	2.14	0.9911	-
5 pulse 45 chase + TritonX	12	3.71	1.97	<0,0001	<0,0001

Panel e - Atg14 RNAi; Syx17 RNAi

**Measured:**	Mean fluorescent intensity of FITC-Avidin per cell (Mean grey value)				
**Statistical test:**	Ordinary one-way ANOVA with Tukey's multiple comparisons test				
**Experiment**	**N**	**Mean**	**Standard deviation**	**P-value** Compared to 5,0	**P-value** Compared to 5,45
5 pulse 0 chase	12	16.70	2.31	-	0.1768
5 pulse 45 chase	12	14.60	4.14	0.1768	-
5 pulse 45 chase + TritonX	12	3.01	1.54	<0,0001	<0,0001

Panel f - Plekhm1 RNAi

**Measured:**	Mean fluorescent intensity of FITC-Avidin per cell (Mean grey value)				
**Statistical test:**	Ordinary one-way ANOVA with Tukey's multiple comparisons test				
**Experiment**	**N**	**Mean**	**Standard deviation**	**P-value** Compared to 5,0	**P-value** Compared to 5,45
5 pulse 0 chase	12	11.70	1.02	-	0.1403
5 pulse 45 chase	12	10.60	1.48	0.1403	-
5 pulse 45 chase + TritonX	12	4.48	1.72	<0,0001	<0,0001

Panel g - Plekhm1 RNAi; Syx17 RNAi

**Measured:**	Mean fluorescent intensity of FITC-Avidin per cell (Mean grey value)				
**Statistical test:**	Ordinary one-way ANOVA with Tukey's multiple comparisons test				
**Experiment**	**N**	**Mean**	**Standard deviation**	**P-value** Compared to 5,0	**P-value** Compared to 5,45
5 pulse 0 chase	12	12.60	2.01	-	0.1077

5 pulse 45 chase	12	10.55	1.96	0.1077	-
5 pulse 45 chase + TritonX	12	4.09	1.70	<0,0001	<0,0001
